# Targeting Picornavirus Nonstructural 2C Protein: Structure, Function, and Antiviral Discovery

**DOI:** 10.1021/acsinfecdis.6c00387

**Published:** 2026-07-27

**Authors:** Kan Li, Guangjin Fan, Wenyi Zhang, Jun Wang

**Affiliations:** Department of Medicinal Chemistry, Ernest Mario School of Pharmacy, Rutgers University, Piscataway, New Jersey 08552, United States

**Keywords:** Picornaviruses, 2C protein, viral replication, morphogenesis, antiviral

## Abstract

Picornaviruses cause substantial human and veterinary disease, yet direct-acting antivirals remain limited. The nonstructural protein 2C is among the most conserved picornaviral proteins and has ATPase and helicase-like activities. 2C is involved in membrane remodeling, RNA replication, encapsidation, morphogenesis, and multiple virus–host interactions. In this review, we summarize the domain architecture and structural biology of 2C, highlighting the N-terminal membrane-binding region, the ATPase core, the C-terminal helical domain, and accessory motifs that regulate oligomerization and ligand recognition. We further review experimentally supported roles of 2C throughout the viral replication cycle and compare known inhibitors, resistance mutations, and emerging design strategies. Collectively, current evidence supports 2C as a tractable yet still underexploited antiviral target for medically important picornaviruses.

## Graphical abstract

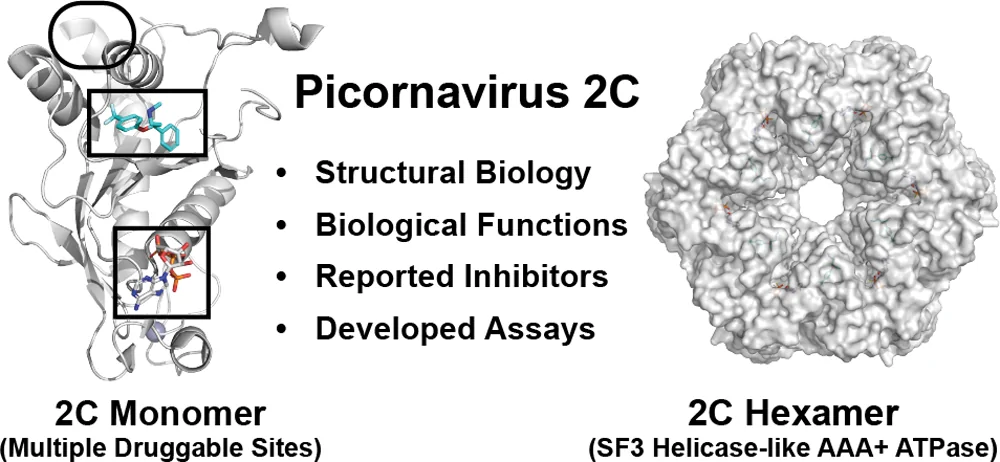

## Introduction

The family *Picornaviridae* is one of the largest and most medically and economically important groups of RNA viruses. According to the current International Committee on Taxonomy of Viruses (ICTV) taxonomy, it comprises 159 species distributed across 68 genera, including *Enterovirus*, *Aphthovirus*, and *Hepatovirus*.^1,2^ Members of the *Picornaviridae* are nonenveloped, positive-sense RNA viruses that cause a wide spectrum of diseases in humans and animals. Clinically important human picornaviruses include enteroviruses (EVs), poliovirus (PV), coxsackieviruses (CVs), rhinoviruses (RVs), and hepatitis A virus (HAV).^3^ These viruses can infect the respiratory tract, the central nervous system, the heart, the eyes, and other organs, thereby imposing a substantial global health burden.^4–6^ Recent outbreaks caused by picornaviruses, particularly the re-emergence of poliovirus in multiple regions worldwide, have highlighted the continued global threat posed by this virus family despite extensive vaccination efforts.^7^ In addition to poliovirus, outbreaks associated with enteroviruses and rhinoviruses continue to impose substantial public health and economic burdens.^8^ Other important members include foot-and-mouth disease virus (FMDV) and encephalomyocarditis virus (EMCV). Beyond causing disease in humans, FMDV triggers devastating outbreaks in livestock, while EMCV presents both zoonotic risk and economic impact due to its broad host range.^9,10^

Picornaviral virions are nonenveloped icosahedral particles composed of the structural proteins VP1, VP2, VP3, and VP4 (Figure 1A).^11–14^ Their genomes consist of single-stranded positive-sense RNA containing one open reading frame (ORF), flanked by a structured 5′-untranslated region (5′UTR) and a 3′UTR followed by a poly­(A) tail (Figure 1B).^15^ The 5′UTR contains a 5′ cloverleaf replication element and an internal ribosomal entry site (IRES) that mediates cap-independent translation.^16–18^ Viral protein genome-linked (VPg, 3B) is covalently attached to the 5′ end of the genomic RNA.^19^ Translation of the ORF yields a single polyprotein that is subsequently processed into the P1, P2, and P3 precursor regions by virus-encoded proteases, including 2A^pro^, 3C^pro^, and 3CD.^20,21^ P1 gives rise to VP1, VP3, and VP0, with VP0 subsequently cleaved into VP2 and VP4.^22–24^ Processing of P2 generates 2A^pro^ and the stable 2BC intermediate, which is further cleaved into 2B and 2C. Processing of P3 yields 3AB and 3CD, followed by formation of the mature nonstructural proteins 3A, 3B (VPg), 3C^pro^, and 3D^pol^ (RNA-dependent RNA polymerase).^25^

**Figure 1:**
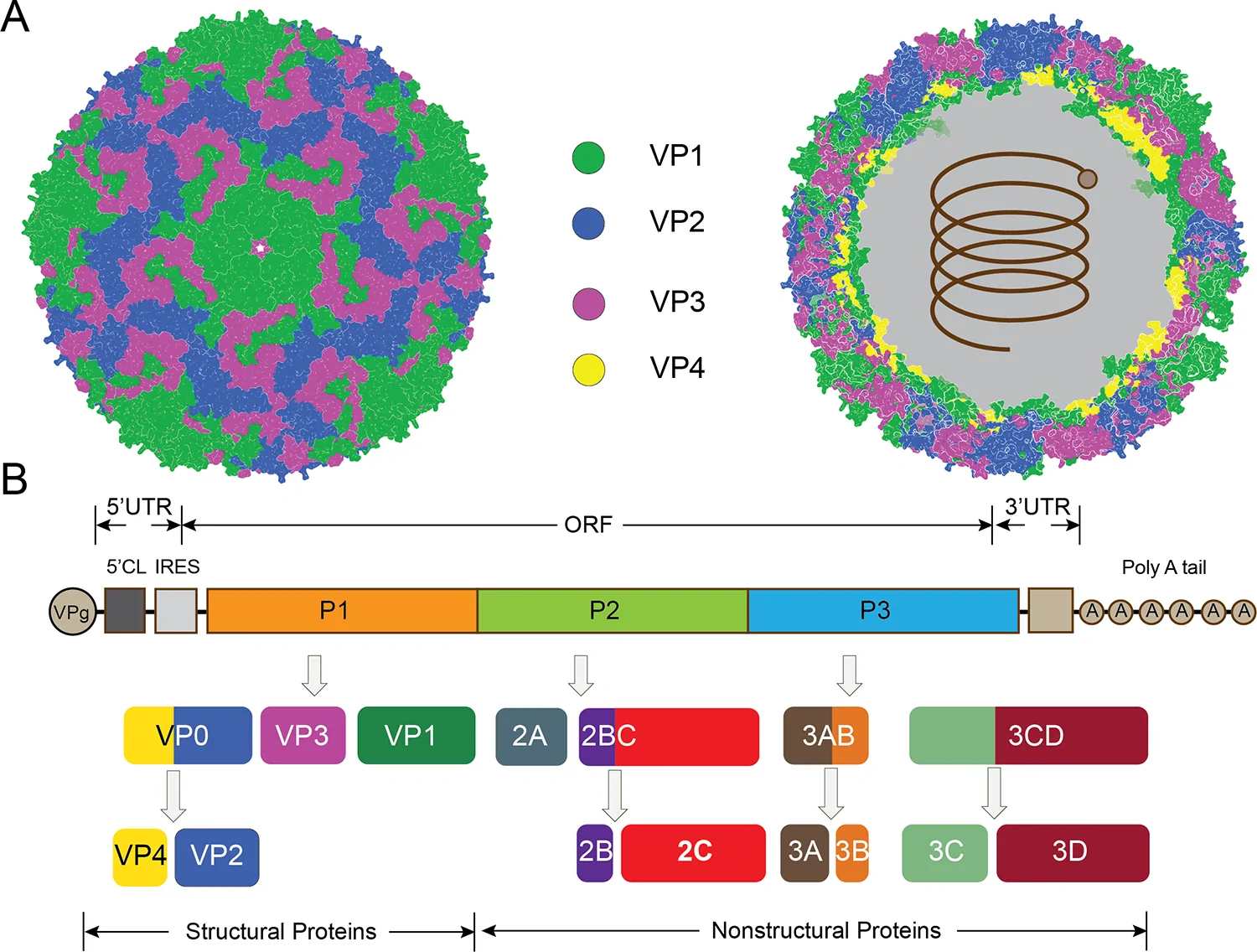
A schematic representation of the viral particle of picornavirus, and the genome organization of the virion with proteolytic processing of the polyprotein, using EV-A71 as a representative. (A) Viral particle structure of EV-A71 (PDB ID: 4AED). (B) The diagram of the genome organization and the polyprotein cleavage of EV-A71 during replication.

Picornaviral RNA replication occurs on virus-induced cytoplasmic replication membranes that are remodeled from host organelles.^26^ Different intracellular membrane sources, including the endoplasmic reticulum, Golgi apparatus, mitochondria, and endolysosomal compartments, can contribute to replication-complex formation.^27–29^ Within this machinery, 2C is one of the most conserved picornaviral proteins and directly contributes to membrane remodeling and replication complex assembly.^25,30–32^ 2C is generally classified as a superfamily 3 (SF3) helicase-like protein and contains conserved ATPase motifs.^25^ Functionally, 2C has been implicated in uncoating, membrane remodeling, RNA binding and replication, encapsidation, and morphogenesis.^25,33^ Its high sequence and structural conservation, and its pleiotropic role in infection, make 2C an attractive target for broad-spectrum antiviral development.^25,34^ For the purpose of this review, broad-spectrum activity is defined as antiviral activity against more than two viral genotypes.

In this review, we summarize the structural biology and biological functions of 2C across the viral replication cycle and survey reported small-molecule and peptide-based 2C inhibitors, with emphasis on their mechanisms of action and drug resistance. Although the conserved nature of 2C strongly supports its potential as a broad-spectrum antiviral target, broad-spectrum antiviral activity has not been uniformly demonstrated across different 2C inhibitor classes or picornavirus species. Meanwhile, there is a significant imbalance in current 2C research, which has predominantly focused on PVs, CVs, and EVs. In contrast, studies investigating 2C proteins and their inhibitors in other clinically relevant picornaviruses, including parechoviruses, cardioviruses, HAV, EMCV, and related species, remain limited. Further investigations are therefore needed to expand the understanding of 2C biology and develop the therapeutic potential of 2C-targeting antivirals across the broader *Picornaviridae* family.

### Structural Biology of 2C

Picornaviral 2C is typically a protein of approximately 330 amino acid residues and contains several domains, including an N-terminal membrane-binding domain, a central ATPase domain, a zinc finger domain in some picornaviruses, an RNA-binding region, and a C-terminal helical segment that contains the pocket-binding motif (Figure 2A).^25,34^ Amphipathic α-helices are present at both the N and C termini and are critical for membrane association, protein tethering, and self-oligomerization.^34^ Overall, 2C shows a high degree of sequence and structural conservation across picornaviruses, with the zinc finger domain representing a notable point of divergence.^25^ Several crystal structures of soluble 2C fragments from PV, EV-A71, CV–B3, FMDV, HAV, and EMCV have now been reported (Table 1), and structural alignment further underscores the structural conservation of 2C across representative picornaviruses (Figure 2B). The EV-A71 2C structures encompassing residues 116–329 (PDB IDs: 5GQ1 and 5GRB) were the first high-resolution 2C structures reported from this family.^35^ PV 2C, however, remains the most extensively studied member, and its 2C crystal structure (PDB ID: 5Z3Q) was reported subsequently in 2018 (Table 1 and Figure 2B).^36^ Together, these structures provide important mechanistic insight into 2C function. These structures implicate a specific 2C–2C interface mediated by the C-terminal helix, providing structural support for a proposed hexameric ring model; however, an experimentally determined hexameric 2C structure remains unavailable.^35–38^ In addition, an allosteric binding pocket for (*S*)-fluoxetine (SFX) was identified in CV–B3 2C and subsequently validated in EV-A71 and EV-D68 2C.^37,39–41^ Although experimental structures are still unavailable for some picornaviruses, including EV-D68 and RV, homology modeling approaches, including AlphaFold3-based modeling in combination with molecular docking and molecular dynamics simulations, have been used to predict 2C–inhibitor interactions.^40^

**Figure 2:**
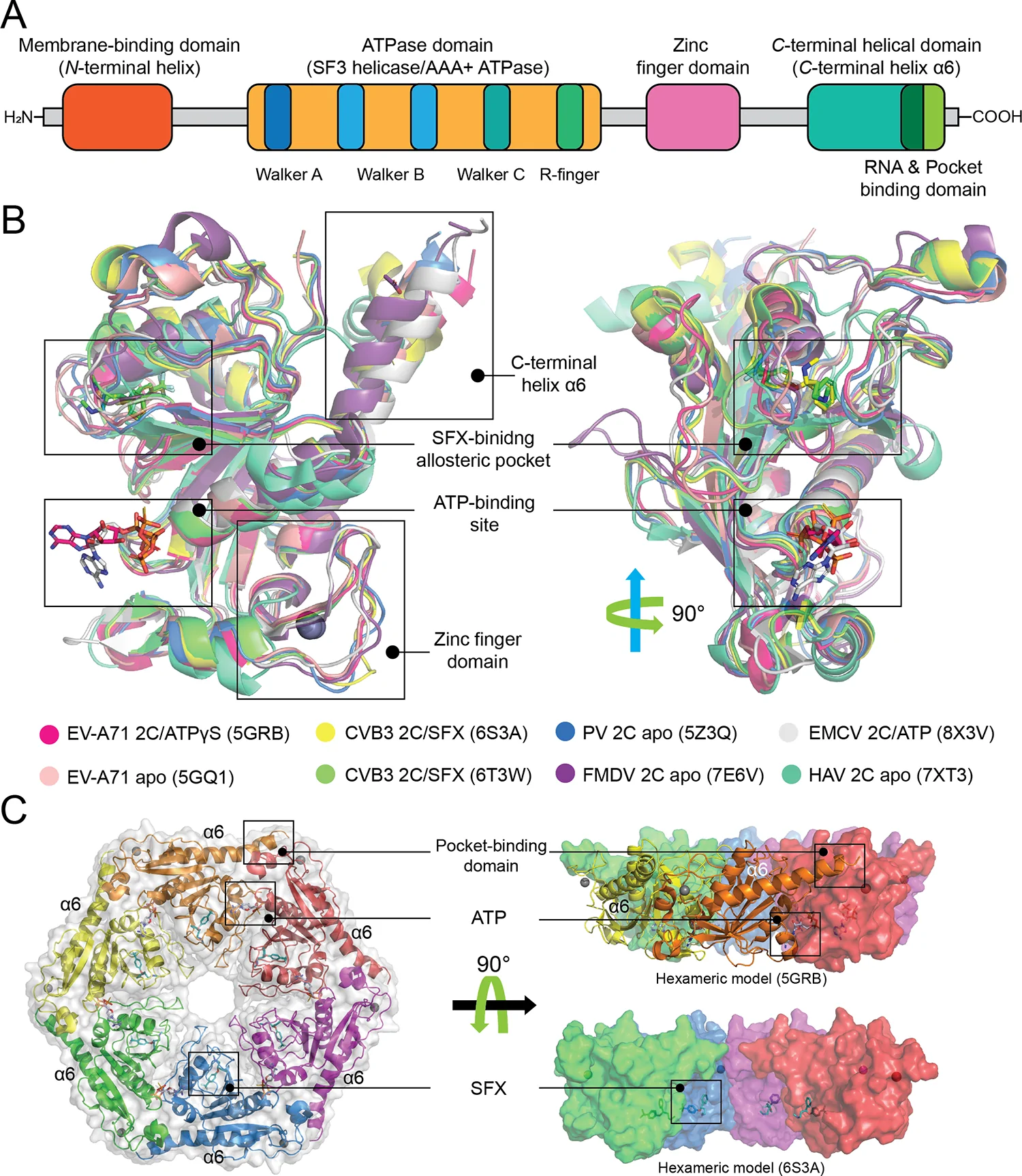
Structures of monomeric 2C proteins and modeled hexameric assemblies. (A) Diagram of major functional motifs in 2C. The protein contains an N-terminal membrane-binding region (AH domain), a conserved superfamily 3 (SF3) helicase-like AAA+ ATPase core, a zinc-binding domain in selected picornaviruses, and a C-terminal helical domain (α6) that includes the RNA-binding segment and PBD. (B) Alignment of reported picornaviral 2C crystal structures. (C) Overview of 2C hexameric models generated based on the JC polyomavirus large T antigen structure (PDB ID: 5J4Y), colored by chain. ATP-γ-S is shown as cyan sticks, and ATP is shown as blue sticks.

**Table 1:** List of the Crystal Structures of Picornavirus 2C Proteins

PDB ID	Structure	2C Length (aa)	Ligand	Resolution (Å)	Refs
5GQ1	EV-A71 2C apo	116–329	-	2.49	^35^
5GRB	EV-A71 2C	116–329	ATPgammaS ATP-γ-S	2.80	^35^
5Z3Q	PV1 2C apo	116–329	-	2.54	^36^
6T3W	CV–B3 2C	117–329	(*S*)-Fluoxetine	1.82	^37^
6S3A	CV–B3 2C	117–329	(*S*)-Fluoxetine	1.52	^37^
7E6V	FMDV 2C apo	97–318	-	1.83	^38^
7XT3	HAV 2C apo	128–335	-	2.15	^42^
8X3V	EMCV 2C	109–325	ATP	2.90	^43^
9RQK	CV-B3 2C	116–329 aa	Compound 53	1.72	^44^

### N-Terminal Membrane-Binding Domain

The N-terminal membrane-binding domain of 2C, commonly described as an amphipathic helix (AH) region, mediates tethering of 2C to the membranes of replication organelles (Figure 2A).^30,45^ In PV, the N-terminal region of 2C contains an amphipathic α-helix that mediates endoplasmic reticulum (ER) membrane targeting and drives membrane reorganization, as demonstrated using chloramphenicol acetyltransferase (CAT) fusion constructs.^30,45–48^ Comparable amphipathic helical elements are conserved across multiple picornaviruses, including EV-A71, CV-A18, and HRV.^45^ Beyond membrane anchoring, the AH domain contributes to membrane remodeling: PV 2C and its N-terminal amphipathic helix can bind lipid droplets and liposomes, and the amphipathic segment can remodel spherical liposomes into tubules.^46,49,50^

A persistent technical challenge in recombinant expression of full-length 2C is its poor solubility and propensity to aggregate, which has limited biochemical and structural characterization.^51,52^ Consequently, the majority of the reported structures use N-terminal truncations lacking the amphipathic helix or a larger portion of the N-terminus (Table 1).^39,40,53,54^ Full-length or engineered hexameric 2C proteins form ring-shaped hexamers with robust ATPase activity, whereas N-terminally truncated constructs exhibit reduced ATPase function.^36,37,51^

### ATPase Domain

The central ATPase domain contains the conserved superfamily 3 (SF3) motifs A, B, and C (Figure 2A).^35,55,56^ Motifs A and B correspond to the Walker nucleotide-binding motifs, whereas motif C is characteristic of SF3 helicase-like proteins.^57,58^ Together with conserved arginine finger residues contributed in trans from a neighboring subunit, these motifs form the ATP-binding and hydrolysis machinery of 2C.^35,59,60^ Mutagenesis within this region strongly impairs ATPase activity and, in systems in which helicase or RNA-remodeling activity has been demonstrated, also disrupts those functions.^35,55,56^ For instance, an endogenous ATP molecule was captured in the crystal structure of EMCV 2C, revealing a unique compact ATP-binding conformation within the catalytic site (Figure 2A,B).^43^ Notably, the γ-phosphate of ATP directly interacts with Arg311, a residue highly conserved on 2C among cardioviruses, and mutation of Arg311 (R311S) markedly reduced ATPase activity but unexpectedly enhanced viral replication.^43^

In oligomeric models of 2C, the catalytic ATP-binding site is positioned between the phosphate-binding loop of one subunit and the arginine finger of the adjacent subunit (Figure 2B, C).^59,60^ In addition to the catalytic site, a distinct allosteric pocket was identified in the high-resolution CV–B3 2C-(*S*)-fluoxetine structure.^37^ This hydrophobic pocket lies between the α2 helix and the Walker B region and is topologically distinct from the catalytic site.^37^ Ligand binding at this site appears to lock 2C into a defined conformation, thereby suppressing the conformational changes required for ATP hydrolysis and productive oligomer function.^37^ The existence of a related allosteric pocket has been supported by structural modeling, mutagenesis, and biochemical studies for EV-A71, EV-D68, and CV–B3.^40,61^

### Zinc Finger Domain and Zinc Finger Equivalent Region (ZFER)

The zinc-binding region is one of the least conserved structural elements of picornaviral 2C. Many enteroviruses, including PV, EVs, RVs, and CVs, contain a cysteine-rich region near the C terminus. Biochemical studies showed that these cysteine-rich motifs bind zinc and function as zinc-coordination sites.^47,62^ In PV, the domain adopts a canonical CCCC-type arrangement, whereas EV-A71 and several other enteroviruses contain a CCC-type motif lacking the second cysteine found in PV.^35,62^ Structural analyses of PV and EV-A71 2C revealed unusual zinc coordination geometries and established this region as an “EV 2C-like” zinc-binding fold distinct from classical zinc-finger families.^35,36^ Functionally, the zinc-binding region contributes to structural stability, oligomerization, and selected protein–protein interactions.^34,36^ Mutational analyses suggest that individual zinc-coordination residues make nonequivalent contributions to 2C folding, hexamer stability, and virus replication.^34,36^ In PV, for example, mutation of Cys272 has been linked to temperature sensitivity and encapsidation defects, highlighting the importance of this region in the enteroviral life cycle.^55,63^

In contrast, corresponding motifs are absent from HAV, FMDV, and EMCV; a region equivalent to enteroviral ZFER superimposes with the corresponding domain (Figure 2A,B and Table 1).^35,36,62,64^ The ZFER is smaller and more compact compared to the zinc finger domain on enteroviral 2C. ZFER lacks the essential cystine residues and plays its roles in 2C structural stability without zinc coordination.^35,36,62,64^

### C-Terminal Helical Domain

Unlike many other AAA+ ATPases, picornaviral 2C contains a long amphipathic C-terminal helix, designated α6 in available structures (Figure 2B).^25^ This helical region mediates a specific 2C–2C interaction that is fundamental to oligomerization, ATPase activity, and virus replication.^35,36^ The C-terminal segment of α6 engages a hydrophobic pocket on a neighboring subunit and is referred to as the pocket-binding domain (PBD) or, in some picornaviruses such as FMDV, the protein-binding loop (PBL).^35,36,38^ Mutational studies in PV and EV-A71 have shown that disruption of this interface abolishes ATPase activity and impairs viral replication.^35,36^ In PV, a short C-terminal RNA-binding segment (residues 312–319) has also been implicated in RNA binding.^56,65^

Ring-like 2C assemblies have been visualized by electron microscopy and supported by modeled hexameric architectures.^35–38,51,52,64^ In these models, the C-terminal helix of one subunit inserts into the neighboring pocket, thereby linking adjacent subunits and stabilizing the hexamer (Figure 2C).^35,37^ However, the C-terminal helix alone is insufficient to generate a native hexamer from truncated ΔN constructs, indicating that both the N- and C-terminal regions contribute to assembly and stability.^36,40,51^ Consistent with this model, fusion of an artificial hexamerization module to the truncated CV–B3 2C-Δ116 produced a soluble hexamer with substantial ATPase activity.^37^

### Functions of 2C in Picornaviruses

The functions of 2C are most clearly understood in the context of the picornaviral life cycle (Figure 3). Although PV has historically served as the principal model for picornavirus biology, studies of nonpolio enteroviruses and related picornaviruses have expanded current understanding of 2C function.^66–68^ After receptor binding and entry, the viral RNA is released into the cytoplasm, VPg is removed, and translation is initiated via the IRES in the 5′UTR. The resulting polyprotein is processed to generate the mature viral proteins required for replication, assembly, and release. Genome replication occurs on virus-induced membranous replication organelles, where VPg primes negative-strand synthesis and newly synthesized positive-strand genomes are subsequently used for translation, further replication, or encapsidation. Within this cycle, 2C functions primarily during the intermediate and late stages of infection, coordinating membrane remodeling, replication complex assembly, RNA metabolism, and morphogenesis.^25,34,66–69^

**Figure 3:**
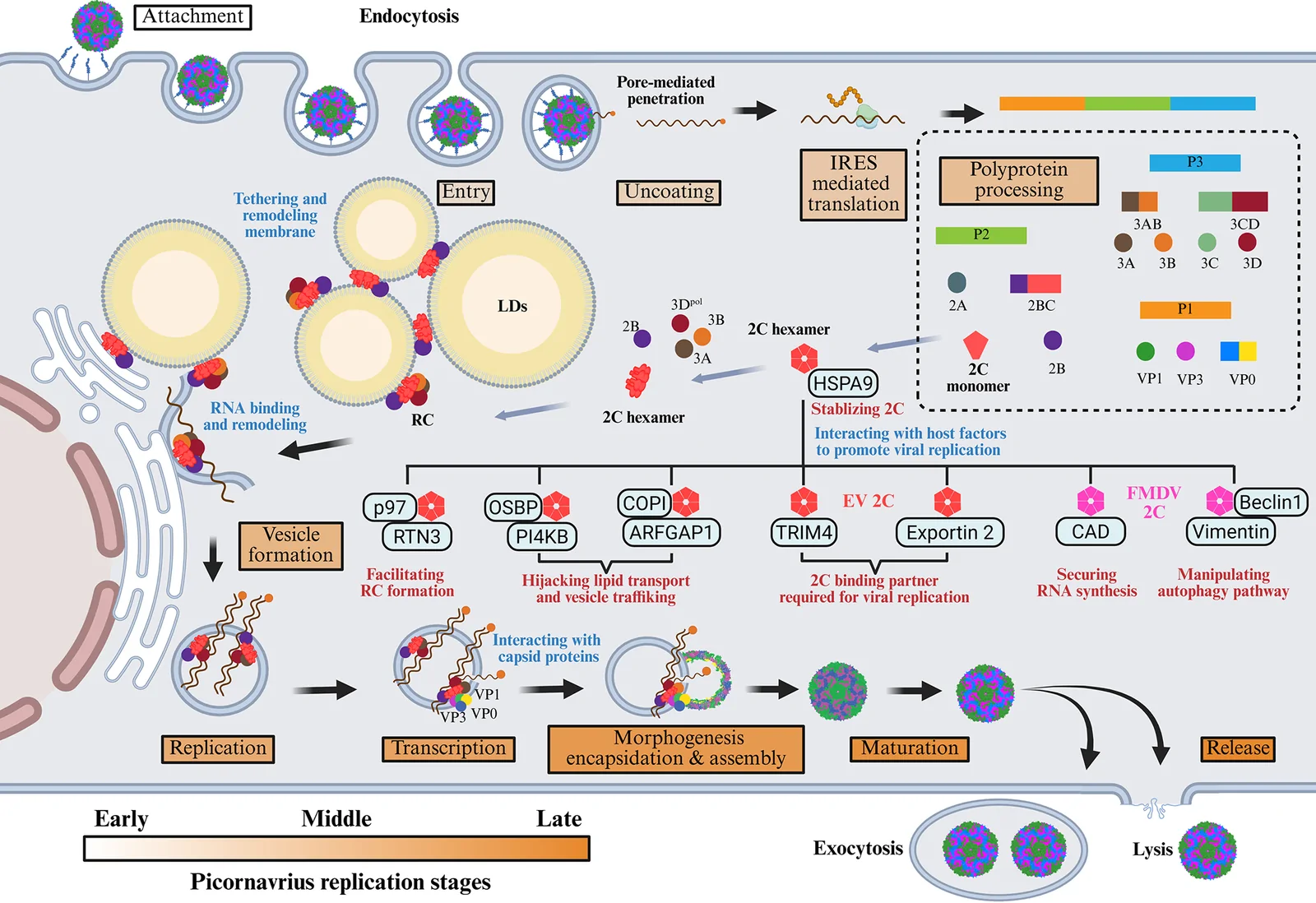
Schematic overview of the picornaviral life cycle and the roles of 2C in replication and morphogenesis. After receptor binding and entry, the viral RNA genome is released into the cytoplasm, where translation is initiated through the IRES in the 5′UTR, and the viral polyprotein is synthesized and processed. During the middle stage of infection, 2C contributes to oligomer formation, replication-organelle biogenesis, membrane remodeling, and RNA replication. During the late stage, 2C also participates in encapsidation and virion morphogenesis through functional interactions with structural proteins. In parallel, 2C engages host factors that promote replication (not all interactions shown). Figure created with BioRender.

### Roles of 2C in Viral Replication

#### Tethering and Remodeling Membranes

2C, together with its precursor 2BC, is a core component of picornaviral replication complexes.^25,34^ It associates with intracellular membranes, promotes the formation of replication organelles, and helps recruit additional viral and host components required for efficient RNA synthesis.^25,34,69,70^ In enteroviruses, 2B and 2C also localize to lipid droplets and contribute to their clustering, consistent with a role in membrane remodeling and lipid mobilization during replication.^50,69,70^ Together, these observations support a model in which 2C connects viral proteins with remodeled host membranes, thereby helping establish the specialized replication organelle where viral RNA synthesis occurs.

#### Interacting with Host Factors

2C also promotes replication through interactions with host factors that regulate membrane architecture, protein homeostasis, and nucleotide supply. A growing body of studies indicates that 2C engages proteins such as HSP70 family members, reticulon 3, p97/VCP, CAD, Beclin1, and vimentin, among others, to enhance replication-organelle biogenesis or to support viral RNA synthesis.^25,34,71–85^ Some proposed host interactions remain incompletely characterized, and in several cases, the precise mechanistic contribution of 2C to these pathways still needs to be defined. Overall, available evidence supports the view that 2C is an important hub for reprogramming host membranes and metabolism to favor viral replication.

#### Binding and Remodeling Viral RNA

In addition to its membrane-associated functions, 2C is involved in viral RNA remodeling. Picornaviral 2C has ATPase-dependent helicase-like activity and ATP-independent RNA chaperoning activity in some systems, and both functions have been linked to efficient genome replication.^25,56,86,87^ Mutations in the conserved ATPase motifs impair RNA synthesis and virus production, underscoring the central role of this domain in the replication cycle.^25,35,56^ These RNA-remodeling activities are thought to facilitate template usage, structured RNA rearrangements, and productive RNA synthesis by the viral polymerase.^25,56^

### Roles of 2C in Morphogenesis and Uncoating

Beyond RNA replication, 2C also contributes to virion morphogenesis and, in some systems, uncoating. Genetic and biochemical studies in PV indicate that 2C interacts functionally with capsid proteins during encapsidation and that mutations in 2C can disrupt particle assembly.^25,55,63,88–91^ Additional evidence links specific PV 2C residues to temperature-sensitive uncoating phenotypes, further supporting a role for 2C in late events of the viral life cycle.^89–92^ Consistent with these findings, the 2C-targeting compound hydantoin inhibits a late stage of PV replication, namely encapsidation.^91^

### Roles of 2C in Immune Response

Picornaviral 2C has also been described as a mediator of virus–host interactions and an important modulator of innate immunity. Through interactions with multiple host factors, 2C suppresses antiviral responses during picornavirus infection.^25,34^ These immune-evasion activities operate through several distinct mechanisms. 2C downregulates major histocompatibility complex class I (MHC I) expression and perturbs vesicle trafficking, thereby impairing antigen presentation to cytotoxic T cells.^93–95^ 2C also targets the cytosolic viral RNA sensors retinoic acid-inducible gene I (RIG-I) and melanoma differentiation-associated protein 5 (MDA5), resulting in their degradation or functional inhibition and thereby reducing interferon-β (IFN-β) production.^96–99^ In addition, 2C suppresses nuclear factor κB (NF-κB) signaling by interfering with IκB kinase (IKK) complexes or preventing assembly of the NF-κB p65/p50 transcriptional complex.^100–103^ 2C further inhibits nucleotide-binding oligomerization domain-containing protein 2 (NOD2)-mediated signaling and promotes degradation of the host restriction factor apolipoprotein B mRNA-editing enzyme catalytic polypeptide-like 3G (APOBEC3G) through the autophagy–lysosome pathway.^104–106^ Although these findings suggest additional opportunities for host-directed antiviral intervention, this topic is beyond the scope of the present review. Key challenges remain, including the need to more completely define 2C interaction networks and to address safety concerns associated with perturbing virus–host interactions. These issues are especially relevant for enteroviruses, which disproportionately affect infants and children and therefore impose a high bar for toxicity and tolerability. Further studies are needed to determine whether 2C-mediated immune modulation can be safely and effectively exploited for therapeutic benefit.

### Inhibitors of 2C against Picornaviruses

Since the identification of guanidine hydrochloride (GuHCl) as a 2C inhibitor, considerable effort has focused on discovering additional picornaviral 2C ligands and inhibitors. Reported 2C inhibitors can be broadly classified into screening-derived hits and synthetic analogs generated through medicinal chemistry optimization. High-throughput phenotypic screening campaigns have yielded several chemically diverse hit series, including 2-(α-hydroxybenzyl)-benzimidazole (HBB), fluoxetine, 2C-2, 2C-4, and dibucaine, which have provided promising starting points for medicinal chemistry optimization (Table 2). Subsequent analog synthesis has expanded these scaffolds and, in some cases, improved antiviral potency. In parallel, advances in 2C structural biology and computer-aided design have enabled structure-based design and virtual screening approaches, which have identified additional hits while reducing the time and experimental cost associated with early stage discovery.

**Table 2:**
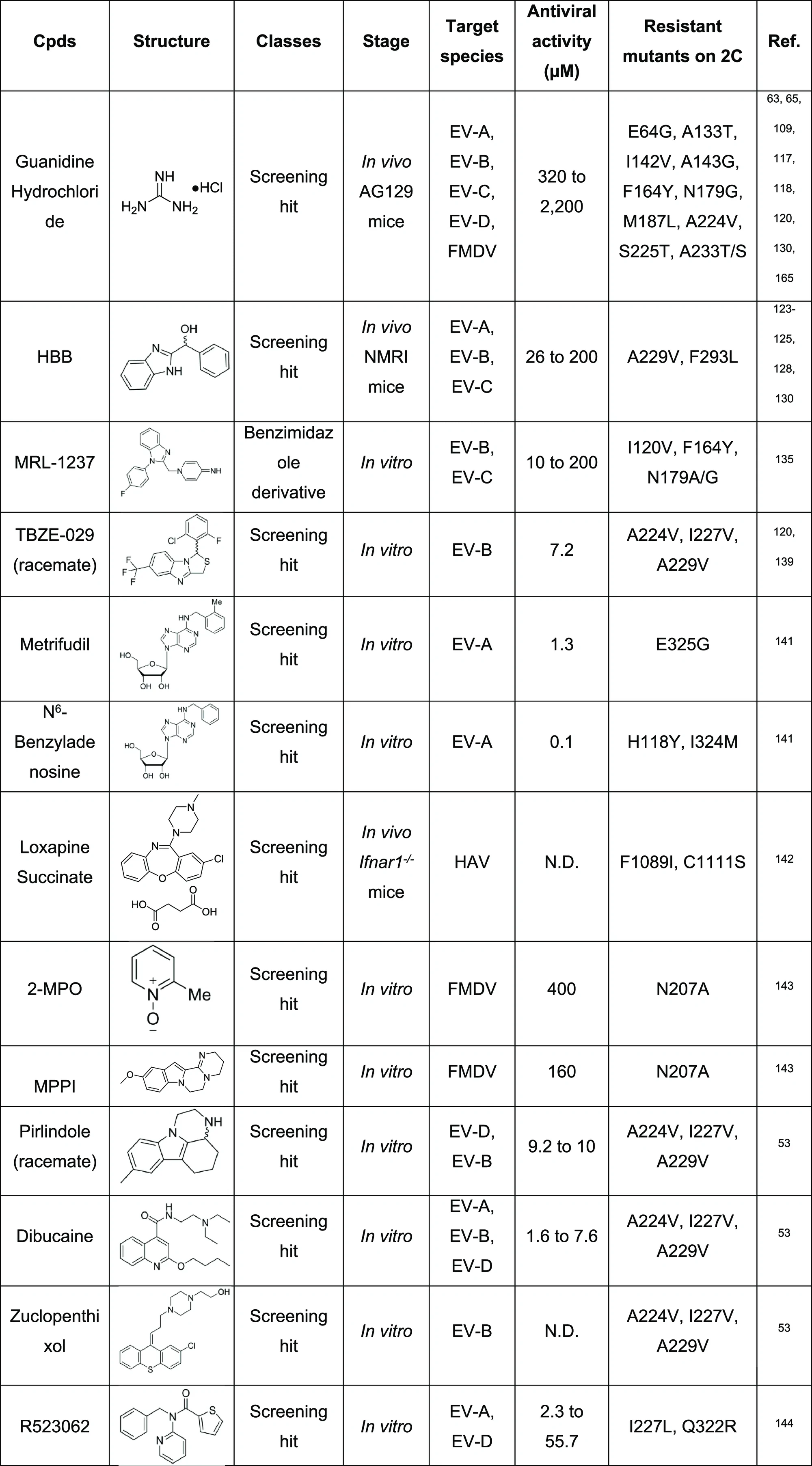

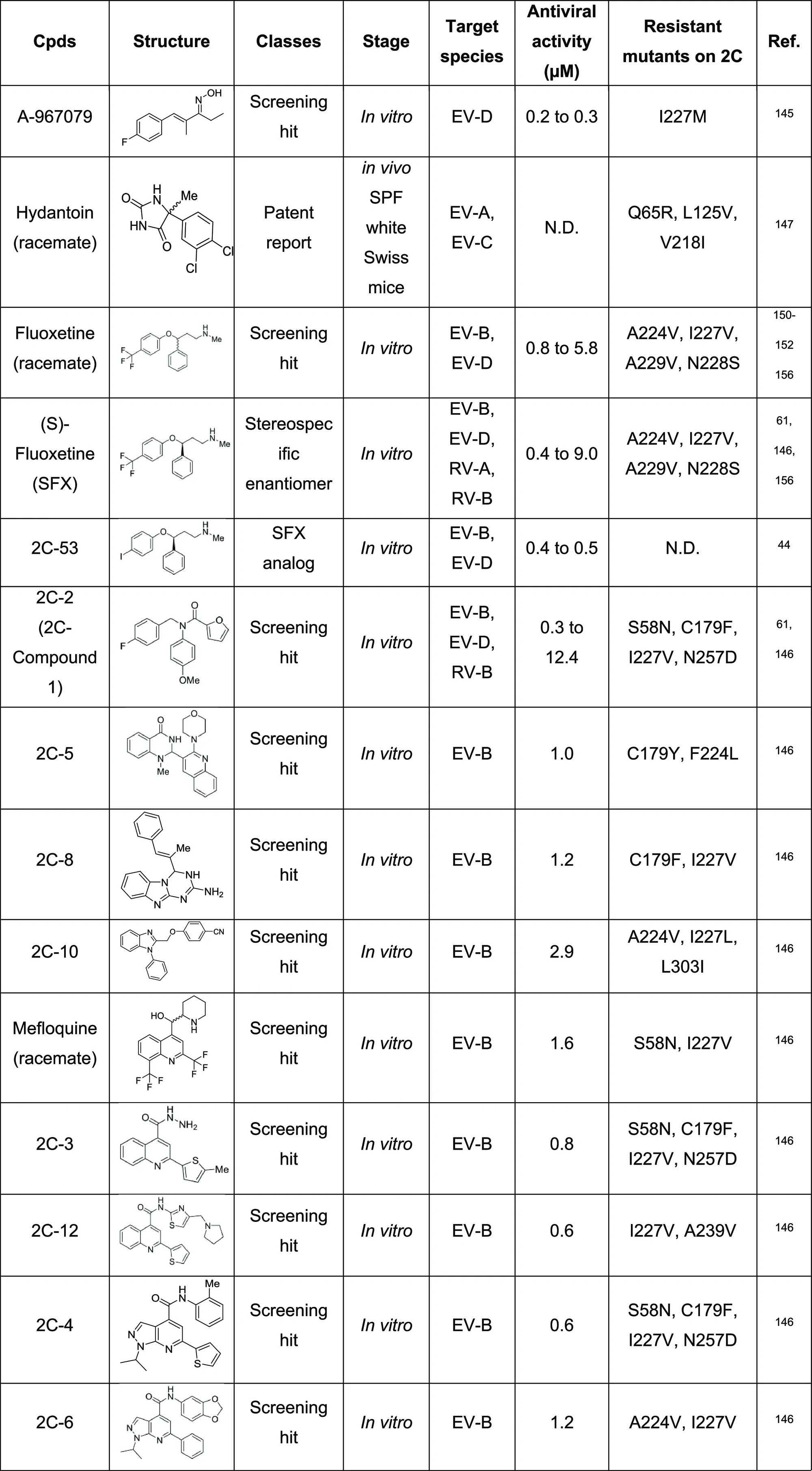

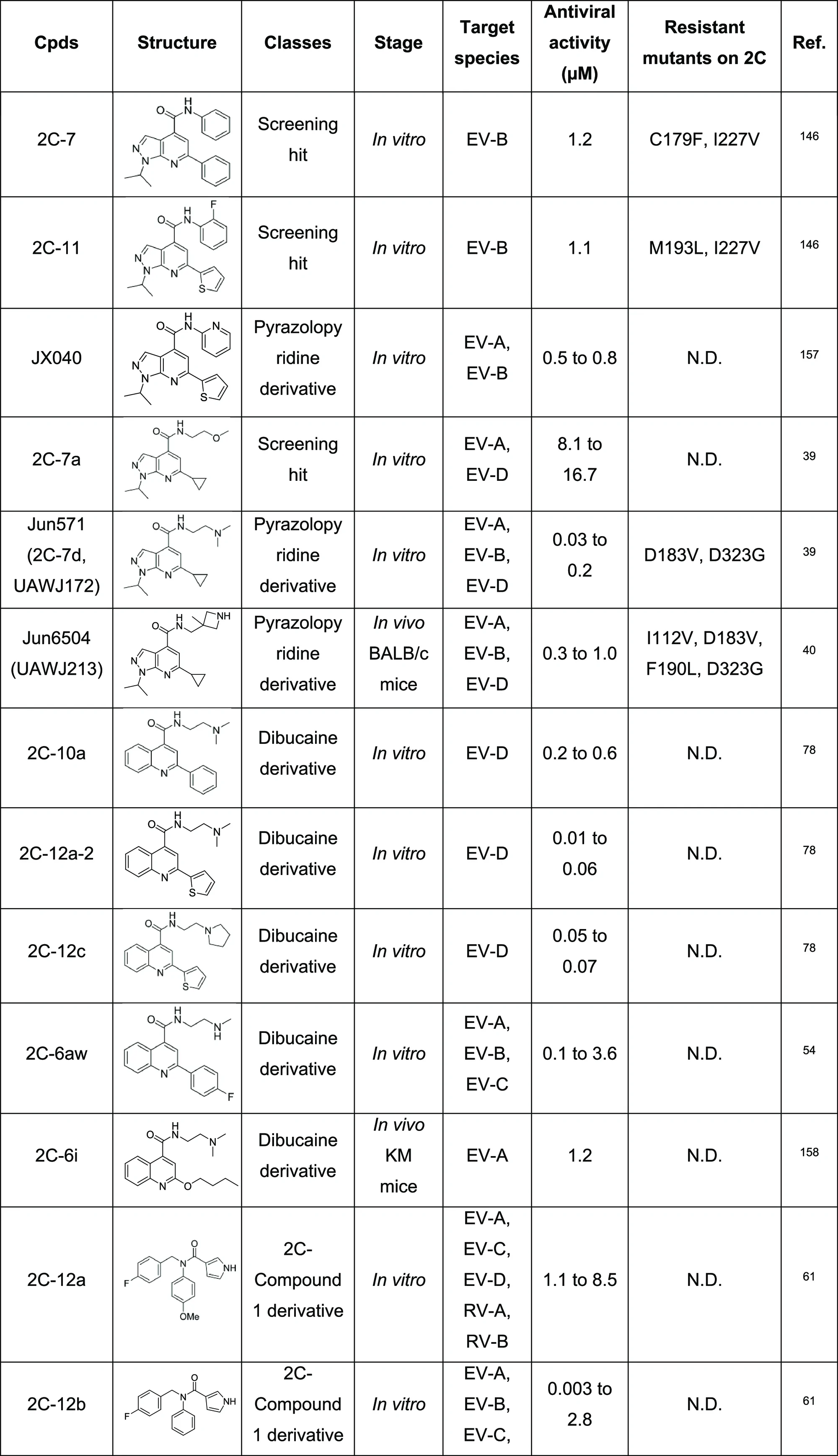

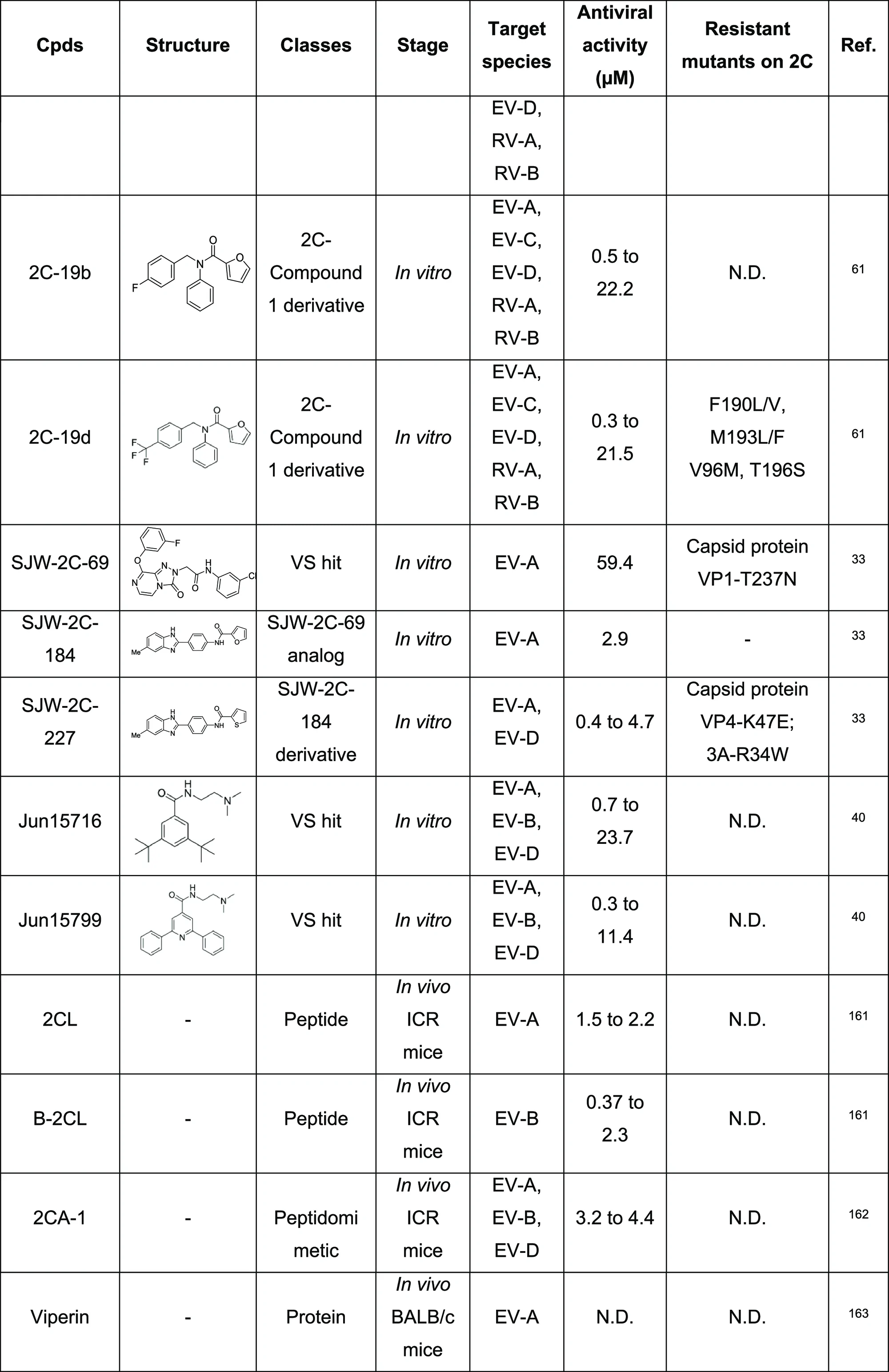
List of Representative 2C Inhibitors against *Picornavirus* Tested *In Vitro* and *In Vivo* ^a^

a N.D. as not determined; dash as not provided on the table; It should be noted that the antiviral assays were performed under different experimental conditions; therefore, direct comparison of EC_50_ values across studies should be interpreted with caution.

Although this review broadly addresses picornavirus 2C inhibitors, the current 2C antiviral discovery landscape is dominated by enteroviruses, particularly EV-A71, CV–B3, and EV-D68. This emphasis largely reflects the greater availability of structural information, biochemical assays, and disease-relevant infection models for these viruses. Accordingly, enterovirus-directed inhibitors constitute most reported 2C-targeting compounds and receive proportionally greater coverage in this section.

Nevertheless, several nonenteroviral 2C inhibitors have recently been reported. These include the FMDV 2C helicase inhibitors 2-MPO and MPPI, identified using a FRET-based screening platform, as well as the repurposed antipsychotic agent loxapine succinate. Some 2C inhibitors have advanced to lead candidates with demonstrated in vivo efficacy in animal infection models, whereas others have primarily served as pharmacological probes to interrogate 2C function. Rationally designed peptides and antiviral proteins have also been described that inhibit viral replication through mechanisms involving 2C.

Rather than comprehensively cataloging all reported picornavirus 2C inhibitors, this review selectively focuses on compounds with relatively well-characterized mechanisms of action and clear translational potential. Natural products, promiscuous compounds, PAINS liabilities, and inhibitors with weak activity or insufficient pharmacological characterization are not discussed in detail.

Below, we summarize representative 2C inhibitors with emphasis on development stage, antiviral spectrum, potency, and resistance-associated mutations (Table 2). Despite the high structural conservation of picornaviral 2C proteins, different inhibitor classes exhibit marked species specificity (Figure 2), underscoring both the promise and the challenge of developing broad-spectrum 2C-targeted antivirals.

### Guanidine Hydrochloride (GuHCl)

GuHCl (Table 2) is an FDA-approved drug used to treat Lambert–Eaton myasthenic syndrome, a rare autoimmune disorder characterized by impaired neuromuscular transmission.^107,108^ Its antipicornaviral activity has been recognized since the 1960s.^109,110^ GuHCl inhibits the replication of a broad range of picornaviruses, including polioviruses (PV; EV-C species), coxsackievirus B3 (CV–B3; EV-B species), echovirus 9 (Echo 9; EV-B species), enterovirus A71 (EV-A71; EV-A species), enterovirus D68 (EV-D68; EV-D species), and foot-and-mouth disease virus (FMDV), with reported potencies ranging from micromolar to millimolar concentrations.^109,111–113^ *In vivo* efficacy has also been demonstrated in both respiratory and neurological AG129 mouse models of EV-D68 infection.^114^ Mechanistic studies initially showed that GuHCl blocks the initiation of viral RNA synthesis,^115^ and subsequent work established 2C as its principal antiviral target.^116^ Resistance mapping further supported this assignment, with resistance-associated substitutions identified in 2C, including F164Y, N179G, and M187L in PV-1;^117–120^ E64G and A133T in Echo 9;^63^ and I127 V and M193L in EV-A71.^121^ Biochemically, GuHCl inhibits the ATPase activity of PV 2C and CV–B3 2C,^37,58^ and it has also been reported to suppress both the NTPase and helicase activities of EV-A71 2C.^56^ Although guanidine-containing analogs have shown antiviral activity against HCV, HIV, and HPV, the relatively high concentrations required for antiviral efficacy limit their translational potential as picornavirus therapeutics.^25,122^

### 2-(α-Hydroxybenzyl)-benzimidazole (HBB)

The racemic mixture of HBB (Table 2), identified through the screening of 75 benzimidazole derivatives, inhibits the replication and cytopathic effects (CPE) of several picornaviruses, including PVs, CVs, and echoviruses with micromolar potency.^123–125^ Among the two enantiomers, d-(−)-HBB ((*R*)-configuration, Figure 4A) exhibits greater antiviral potency and a higher selectivity index than l-(+)-HBB ((*S*)-configuration).^126^ Although HBB inhibits viral RNA synthesis through a mechanism similar to GuHCl, studies have revealed a distinct antiviral spectrum and only partial cross-resistance between the two inhibitors.^111^ A synergistic antiviral effect has been observed when HBB and GuHCl are used in combination to inhibit picornavirus replication.^127^ *In vivo*, HBB monotherapy successfully protected mice infected with CV-A9, while combined HBB and GuHCl treatment conferred significant protection against Echo 9 and CV-A9-induced paralysis and mortality in newborn Naval Medical Research Institute (NMRI) mice.^128^ Mechanistic studies identified an HBB-resistant strain of Echo 9, with the resistance mapped to the 2C protein, the same target as GuHCl.^63,129,130^ Unlike GuHCl, where resistance is conferred by E64G and A133T on 2C of Echo 9, HBB resistance is driven by a single point mutation, A229V (C4782U), in Echo 9.^63,130^

**Figure 4:**
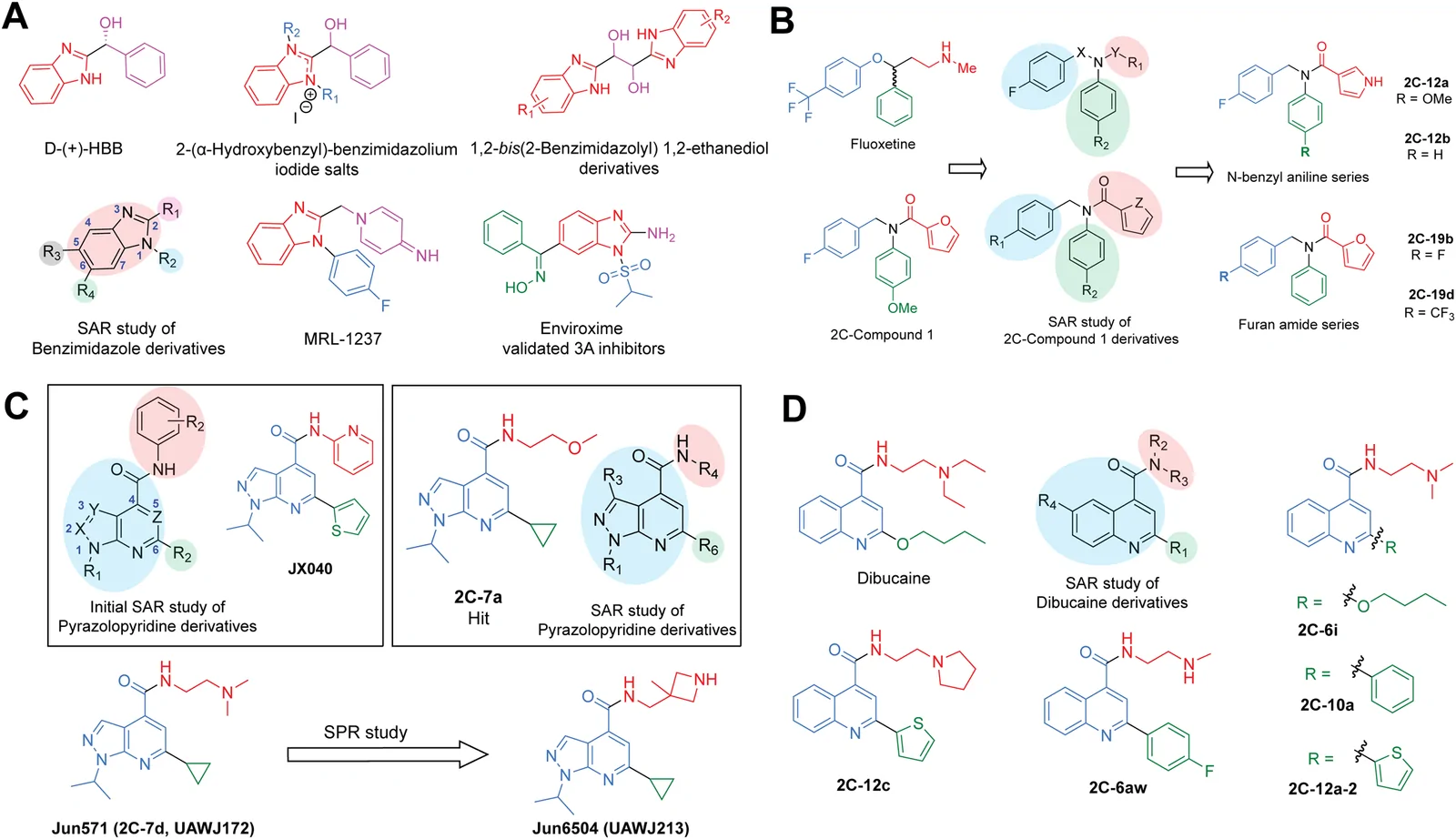
Identification of 2C inhibitors through lead optimization. (A) HBB analogs and other benzimidazole derivatives as 2C inhibitors. (B) SAR study of 2C-Compound 1 and its derivatives as 2C inhibitors. (C) Optimization of pyrazolopyridine derivatives as 2C inhibitors. (D) SAR study of dibucaine and its derivatives as 2C inhibitors.

### Benzimidazole Derivatives as 2C Inhibitors

HBB was a milestone discovery because it was among the first synthetic compounds to selectively inhibit enterovirus replication.^131^ Subsequent analogs were evaluated against PV-2 and echovirus 6. Early SAR studies identified the 2-hydroxybenzyl substituent on the benzimidazole core as an important determinant of selective anti-PV activity, because replacement with hydroxymethyl, α-hydroxyethyl, benzyl, or benzoyl groups diminished potency and selectivity.^131^ 2-(α-Hydroxybenzyl)­benzimidazolium iodide salts (Figure 4A) were later prepared to improve aqueous solubility, but the corresponding quaternary iodides showed markedly reduced selectivity relative to HBB.^132^ Symmetrical 1,2-bis­(2-benzimidazolyl)-1,2-ethanediol analogs (Figure 4A) were subsequently reported as PV inhibitors despite lacking the hydroxybenzyl substituent.^133^ More comprehensive SAR studies further showed that selective antiviral activity depends not only on the hydroxybenzyl group but also on the benzimidazole nitrogens, the overall geometry between the benzimidazole and the para-phenyl ring, the electron-withdrawing character of the scaffold, and the relative orientation of the planar moieties (Figure 4A).^134^

A distinct benzimidazole series was later designed and evaluated, with MRL-1237 emerging as the lead compound (Table 2 and Figure 4A).^134^ MRL-1237 inhibited multiple enteroviruses, including PV-1 Mahoney, attenuated Sabin PV strains, CV–B3 Nancy, and CV–B4 JVB, with EC_50_ values ranging from 0.6 to 11 μM.^135^ Serial passaging and reverse-genetics studies mapped resistance primarily to the 2C region, where the F164L substitution, together with changes at residues I120 and N179, conferred resistance in PV.^135^ MRL-1237 also exhibited an antiviral spectrum and cross-resistance profile similar to those of GuHCl but distinct from those of enviroxime (Figure 4A).^135^ Although enviroxime was initially grouped with substituted benzimidazoles and other putative 2C inhibitors such as GuHCl and HBB,^136,137^ it was subsequently validated as a 3A inhibitor and is therefore not discussed further here.^138^

### TBZE-029

TBZE-029 (Table 2), a thiazolobenzimidazole originally developed as a non-nucleoside reverse transcriptase inhibitor (NNRTI) for human immunodeficiency virus type 1 (HIV-1), was identified in a large-scale antiviral screen against coxsackievirus B (CV–B) strains using a multicycle viral growth assay.^139,140^ TBZE-029 displayed potent antiviral activity against multiple CV–B serotypes, including CV–B3, as well as echoviruses. Unlike HIV-1, TBZE-029 does not target the picornaviral polymerase. Instead, resistance-mapping studies support 2C as its antiviral target.^120^ Three resistance-associated substitutions, A224V, I227V, and A229V, were identified in TBZE-029-resistant CV–B3 variants, with I227V and A229V having the strongest effects on resistance.^120^ These residues map to the conserved 224-AGSINA-229 loop, a recurrent hotspot for resistance to TBZE-029 and several other 2C-directed inhibitors. Consistent with a shared binding or resistance landscape, TBZE-029-resistant viruses also exhibited cross-resistance to other 2C inhibitors, including GuHCl, HBB, and related benzimidazole analogs.^120^

### Metrifudil and N^6^–Benzyladenosine

Metrifudil (Table 2), an A2 adenosine receptor agonist, and N6-benzyladenosine (Table 2), an A1 adenosine receptor agonist, were identified as active hits against EV-A71 in an HTS campaign. The screening campaign used a pharmacologically annotated compound library together with EV-A71 pseudoviruses encapsidating luciferase-encoding replicons.^141^ N6-Benzyladenosine displayed greater potency than metrifudil against EV-A71 pseudoviruses, with EC_50_ values of 0.1 and 1.3 μM, respectively. Both compounds exhibited broad-spectrum antiviral activity against multiple EV-A71 genotypes but were inactive against PV.^141^ Subsequent studies identified 2C as the molecular target of both compounds. Resistance to metrifudil was associated with a single E325G substitution in EV-A71 2C, whereas resistance to N6-benzyladenosine mapped to the H118Y and I324M substitutions.^141^

### Loxapine Succinate

Loxapine succinate, a selective dopamine receptor D2 antagonist, was identified through a phenotypic HTS using a NanoLuc luciferase (Nluc)-expressing HAV (HAV/NLuc) based reporter assay.^142^ Follow-up studies showed that loxapine succinate inhibited viral RNA replication without suppressing viral entry or internal ribosome entry site (IRES)-dependent translation. Serial passage and viral sequencing resulted in the accumulation of viruses containing mutations in the 2C-encoding region, specifically F1089I (F2I on HAV 2C) and C1111S (C24S on HAV 2C) at the N-terminus of 2C.^142^ Animal studies demonstrated that administration of loxapine succinate to HAV-infected *Ifnar1* ^–/–^ mice (which lack the type I interferon receptor) led to a decrease in the levels of fecal HAV RNA and of intrahepatic HAV RNA at an early stage of infection.^142^ This study provided novel insights into antiviral discovery for the treatment of hepatitis A infection.

### 2-MPO and MPPI

Addressing the lack of reported 2C inhibitors against FMDV, a fluorescence resonance energy transfer (FRET)-based HTS platform was established to identify inhibitors targeting the helicase activity.^143^ Screening of 4424 compounds identified 6 hits that inhibited helicase activity by more than 60% at 20 μM. Molecular docking suggested that these compounds interact with the catalytic core of 2C through hydrogen bonding, hydrophobic interactions, and π-cation interactions centered around residue N207. Kinetic analyses classified 2-MPO and MPPI as mixed-type inhibitors (Table 2), whereas other hits exhibited competitive or noncompetitive inhibition mechanisms. Among them, 2-MPO and MPPI demonstrated the most promising antiviral activity against FMDV in indirect immunofluorescence, RT-qPCR, and plaque reduction assays. NanoDSF experiments further supported direct target engagement and revealed that site-directed mutagenesis at N207 on FMDV 2C significantly attenuated compound binding. MPPI effectively suppressing FMDV replication at 160 μM, stronger than 2-MPO, which required concentrations of approximately 400 μM to achieve comparable antiviral effects (Table 2). Although their antiviral activities remained modest, the study established the feasibility of directly targeting the helicase function of 2C and highlighted FRET-based helicase assays as a valuable platform for future FDMV 2C inhibitor discovery.

### Pirlindole, Dibucaine, and Zuclopenthixol

Additional 2C-targeting hits were identified in a separate drug-repurposing screen of the Prestwick Chemical Library, a collection of 1,200 approved drugs.^53^ In this study, HeLa R19 cells were infected with a GFP-expressing CV–B3 Nancy reporter virus (CV–B3-GFP), and viral replication was quantified by measuring GFP fluorescence at 6 h postinfection.^53^ Five compounds, pirlindole, fluoxetine, dibucaine, zuclopenthixol, and formoterol, inhibited CV–B3 replication (Table 2).^53^ Follow-up characterization showed that dibucaine, pirlindole, and zuclopenthixol were most active against EV-B and EV-D viruses, with dibucaine also retaining activity against EV-A, whereas formoterol inhibited all enteroviruses and rhinoviruses tested.^53^ Resistance mapping further showed that the 2C substitutions A224V, I227V, and A229V conferred resistance to dibucaine, pirlindole, and zuclopenthixol, but not to formoterol.^53^ In addition, isothermal titration calorimetry confirmed direct binding of dibucaine to CV–B3 2C *in vitro*, whereas binding to the A224V/I227V/A229V triple mutant protein was reduced.^53^

### R523062 and A-967079

Our group reported novel antipicornavirus compounds targeting 2C. R523062 and A-967079 were validated as 2C inhibitors through a systematic mechanism-deconvolution workflow.^144,145^ Both compounds were identified in a phenotypic screening campaign that combined cell-based cytopathic effect screening of EV-D68 US/MO/14–18947 in RD cells with follow-up antiviral profiling, spectrum testing, time-of-addition experiments, serial passaging, and reverse-genetics validation. R523062 (Table 2), which is structurally related to the previously reported hit 2C-2 (2C-compound 1), inhibited multiple EV-A and EV-D strains with EC_50_ values ranging from 2.3 to 55.7 μM.^61,144,146^ A-967079 (Table 2), which contains a ketoxime moiety, displayed potent and selective activity against contemporary EV-D strains with submicromolar EC_50_ values. Both R523062 and A-967079 inhibited enteroviral RNA and protein synthesis. Time-of-addition experiments indicated that both inhibitors act during the intermediate-to-late stages of replication (1–7 hpi) rather than during viral entry (0–1 hpi). Resistance mapping by serial passaging and reverse genetics identified I227L and Q322R as resistance substitutions for R523062 and I227M, respectively, in A-967079.^144,145^

### Hydantoin

Although its original development history remains unclear, DL-5-(3,4-dichlorophenyl)­methylhydantoin (hydantoin, Table 2) was previously described as an anti-influenza agent and as a treatment for audiogenic seizures in mice. In 1974, Eli Lilly reported hydantoin as an antiviral for CV-A21 and PV-1.^147^ The compound also protected SPF white Swiss mice from CV-A21-induced paralysis and mortality *in vivo*.^147^ L-hydantoin was more potent than DL-hydantoin, requiring 2.5 and 5 μg/mL, respectively, to achieve a 50% reduction in CV-A21 plaque formation.^147^ The mechanism of action of hydantoin remained controversial in the early 2000s due to limited understanding of 2C’s multiple roles in RNA replication and viral morphogenesis.^120,148^ Early studies suggested that hydantoin acts differently from other reported 2C inhibitors such as GuHCl. Specifically, hydantoin did not inhibit RNA synthesis but instead blocked virion assembly at a late stage of the replication cycle, downstream of the step inhibited by GuHCl.^91^ However, resistant viruses were isolated, and site-directed mutagenesis identified three 2C substitutions, Q65R, L125V, and V218I, that conferred resistance to hydantoin.^91^ Later studies further suggested that hydantoin blocks postsynthetic cleavages during PV replication.^149^ A subsequent study published in 2016 reconciled these observations by showing that hydantoin inhibits enterovirus replication through dual mechanisms: at low concentrations, it primarily blocks viral morphogenesis, whereas at high concentrations, it inhibits RNA synthesis.^148^

### SFX and Its Derivatives

Fluoxetine (Table 2), a selective serotonin reuptake inhibitor (SSRI), was identified as a potent CV–B3 inhibitor in multiple drug-repurposing screens.^150–152^ One campaign used a modified CPE assay with an ATP/luminescence readout to screen the Prestwick Chemical Library, whereas another used a Renilla luciferase-expressing CV–B3 reporter virus (CV–B3-Rluc) to screen the NIH Clinical Collection.^150–152^ Fluoxetine is structurally related to TBZE-029 and displayed a similar antiviral spectrum, with preferential activity against EV-B and EV-D species at submicromolar concentrations.^120,152^ Resistance to fluoxetine mapped to three substitutions in 2C, A224V, I227V, and A229V, within the conserved 224–229 AGSINA loop previously implicated in TBZE-029 resistance.^120,151^ However, the antiviral activity of fluoxetine did not translate *in vivo*, as no efficacy was observed in EV-D68 mouse models.^114,153^ Consistent with these findings, clinical studies in patients with acute flaccid myelitis (AFM) showed no therapeutic benefit, although fluoxetine treatment was well tolerated.^154^ The reasons for this lack of efficacy remain unclear. Notably, a single case report described clinical stabilization in an immunocompromised pediatric patient with chronic enteroviral encephalitis, suggesting that context-dependent effects may exist.^155^

Fluoxetine was initially identified and validated as a racemic mixture, but subsequent studies showed that enterovirus inhibition is stereospecific.^156^ The active (*S*)-enantiomer, (*S*)-fluoxetine (SFX, Table 2), exhibited an approximately 5-fold lower EC_50_ (i.e., greater potency) than the racemic mixture.^156^ Homology modeling and structure-guided mutagenesis further identified the resistance substitutions C179F/Y and F190L, providing additional support for 2C as the antiviral target of SFX.^156^

The identification of fluoxetine as a 2C inhibitor represents a notable achievement in structure-based antiviral drug design, as it provides a clinically precedented, drug-like scaffold for subsequent medicinal chemistry optimization. SAR studies of SFX improved both potency and selectivity. Guided by rational design, X-ray crystallography, and computational docking, a series of SFX analogs targeting the allosteric site in the CV–B3 2C ATPase domain was developed and validated in biochemical and cell-based assays. Among these analogs, compound 2C-53 emerged as a potent inhibitor, with EC_50_ values of 0.5 μM against CV–B3 and 0.4 μM against EV-D68, and with improved selectivity relative to SFX (Table 2), representing one of the most advanced 2C inhibitors.^44^

### 2C-2 to 2C-8, 2C-10 to 2C-12, and Mefloquine

Using the HTS strategy that led to the identification of fluoxetine, a subsequent large-scale screen was conducted against 15 representative enteroviruses spanning the EV-A, EV-B, and EV-C species, using six bioactive compound libraries comprising 85,585 small molecules.^146^ Following primary screening, hit validation, and secondary assays, 12 structurally diverse compounds with submicromolar antienteroviral activity were identified.^146^ Of these, 10 compounds, designated 2C-2 to 2C-8 and 2C-10 to 2C-12 (Table 2), were confirmed to target 2C by serial passaging and sequencing of resistant variants.^146^ The antimalarial mefloquine also exhibited antienteroviral activity, but further evaluation was limited by cytotoxicity.^146^ The confirmed 2C-targeting hits encompassed multiple chemotypes: 2C-2, 2C-5, and 2C-8 each featured distinct core scaffolds; 2C-10 was a benzimidazole derivative; 2C-3 and 2C-12 shared a 2,4-disubstituted quinoline scaffold; and 2C-4, 2C-6, 2C-7, and 2C-11 contained a pyrazolopyridine core bearing an isopropyl substituent on the 1H-pyrazole ring.^146^ Resistance profiling in CV–B3 showed that serial passaging with 9 of the 10 compounds selected the I227V substitution in 2C.^146^ In contrast, resistance to 2C-5 mapped to two alternative substitutions, C179Y and F244L.^146^ These hits have since served as useful starting points for 2C-directed antiviral optimization, including analogs of 2C-compound 1 (2C-2) as well as pyrazolopyridine and quinoline series (Table 2).

### Pyrazolopyridine Derivatives as 2C Inhibitors

After pyrazolopyridine-based hits such as 2C-4, 2C-6, 2C-7, and 2C-11 (Table 2) were identified as 2C inhibitors in HTS campaigns, follow-up series were designed and evaluated against PV-1, EV-A71, and CV–B3.^157^ A comprehensive SAR study identified JX040 as the lead compound, with submicromolar EC_50_ values of 0.5–0.8 μM against EV-A71 and CV–B3 but no detectable activity against PV-1 (Figure 4C).^157^

Our group also identified a pyrazolopyridine hit, 2C-7a (Table 2 and Figure 4C), bearing a neutral methyl ether at the 4-amide substituent and showing moderate antiviral activity against both EV-A71 (EC_50_ = 8.1 μM) and EV-D68 (EC_50_ = 16.7 μM).^39^ A systematic SAR campaign subsequently diversified the alkyl substituents at R1 and R3 (blue, Figure 4C), the 4-amide group at R4 (red, Figure 4C), and the aliphatic moiety at R6 (green, Figure 4C). Replacement of the neutral ether at the R4 amide with a basic dimethylamino substituent yielded Jun571 (Figure 4C and Table 2) as the most potent lead in this series. Jun571 inhibited EV-D68, EV-A71, and CV–B3 with EC_50_ values of 70, 100, and 200 nM, respectively, and showed a selectivity index greater than 500.^39^ Mechanistic studies demonstrated that Jun571 binds directly to EV-D68 2C, and resistance mapping by serial passaging and reverse genetics identified D183 V and D323G as resistance substitutions.^39^

Starting from Jun571, a translational advancement toward a preclinical antiviral candidate was pursued (Figure 4C).^40^ Because no EV-D68 2C crystal structure was available, homology modeling and molecular dynamics simulations were used to propose binding of Jun571 to a conserved allosteric pocket corresponding to the S-fluoxetine-binding site identified in CV–B3 2C (Figure 2B).^35,37^ Guided by this model, structure–property relationship studies then targeted improved metabolic stability by replacing the N,N-dimethylethane-1,2-diamine group. Jun6504 (Figure 4C and Table 2), which contains an azetidine moiety, displayed potent antiviral activity against EV-D68 (EC_50_ = 250 nM), high metabolic stability (*t*_1/2_ > 145 min), and favorable *in vivo* pharmacokinetic properties.^40^

Importantly, Jun6504 also demonstrated favorable *in vivo* pharmacokinetic properties, including sustained systemic exposure and improved stability relative to earlier analogs, supporting its suitability for *in vivo* efficacy studies.^40^ After confirmation of broad antienteroviral activity and 2C target engagement, Jun6504 was evaluated in a neonatal mouse model of EV-D68 infection. In this model, neonatal mice were infected with the EV-D68 strain that induces progressive limb paralysis, thereby recapitulating key features of EV-D68-associated acute flaccid myelitis. Treatment with Jun6504 significantly protected mice against virus-induced paralysis and improved survival compared with vehicle-treated controls.^40^ In addition to reducing paralysis severity, antiviral efficacy was associated with decreased viral titers in relevant tissues, supporting a direct antiviral mechanism *in vivo*. Collectively, Jun6504 represents a promising preclinical candidate and provides preclinical proof of concept for targeting 2C as a viable antiviral strategy against EV-D68.^40^

### Dibucaine Derivatives as 2C Inhibitors

In independent validation of reported 2C-targeting hits, dibucaine (Figure 4C and Table 2), with a selectivity index (SI) of 10.7, was chosen as a starting point for SAR exploration due to its modular synthetic accessibility. To improve potency and selectivity, our group synthesized more than 100 quinoline analogs across two studies.^54,78^ Optimization focused on three regions: (1) modification of the R1 position with alkoxy or aryl substituents (green, Figure 4D); (2) variation of the R2 and R3 substituents with linear, cyclic, branched, or aromatic diamines (red, Figure 4D); and (3) substitution at the 6-position with F, Cl, or methoxy groups (blue, Figure 4D). Among the first 60 analogs, three lead compounds, 2C-10a, 2C-12a-2, and 2C-12c, showed substantially improved potency against multiple EV-D68 strains, with EC_50_ values below 1 μM and SI values above 180.^78^ A subsequent study of 43 derivatives identified 2C-6aw as a promising lead candidate, with an EC_50_ of 0.2 μM, an SI of 162, and excellent metabolic stability in mouse microsomes (*t*_1/2_ = 114.7 min).^54^

Dibucaine-derived inhibitors with activity against EV-A71 were also reported. Among these compounds, 2C-6i (Figure 4D and Table 2), identified through systematic SAR studies, showed promising potency against EV-A71, with an EC_50_ of 1.3 μM.^158^ *In vivo* studies further showed that 2C-6i reduced clinical symptoms and mortality in infected mice.^158^ Mechanistic studies indicated that 2C-6i targets the 2C helicase and disrupts viral RNA remodeling and metabolism.^158^ Notably, the lead compounds 2C-10a, 2C-12a-2, and 2C-6i all share an *N*,*N*-dimethylethane-1,2-diamine motif in the R2/R3 region. In addition, 2C-10a, 2C-12a-2, 2C-12c, and 2C-6aw each contain an aromatic substituent at the 2-position of the quinoline core. These recurring structural features were also incorporated into subsequent pyrazolopyridine analogs and provided useful guidance for further optimization of 2C inhibitors.

### 2C-Compound 1 Derivatives as 2C Inhibitors

2C-compound 1 (Table 2 and Figure 4B), which was initially identified by HTS, is structurally related to fluoxetine but lacks a chiral center, making it an attractive lead for further optimization. Two derivative series were subsequently developed by dividing the scaffold into three regions: one derived from the furan amide moiety and two derived from the N-benzylaniline moiety (Figure 4B). Among the representative analogs, 2C-12a, 2C-19b, and 2C-19d (Table 2 and Figure 4B) displayed improved antiviral activity against multiple enterovirus and rhinovirus species, along with reduced cytotoxicity, relative to the parent 2C-compound 1. Serial passaging with 2C-19d followed by analysis of resistant EV-A71, CV–B3, and EV-D68 variants identified 2C resistance mutations that also conferred cross-resistance to 2C-compound 1, 2C-12a, and 2C-19d (Table 2). By combining the structural features of 2C-12a and 2C-19b, 2C-12b (Table 2 and Figure 4B) emerged as the most potent broad-spectrum inhibitor, with activity against EV-A71, CV–B3, PV-1, CV-A24, EV-D68, HRV-A2, and HRV-B14 and EC_50_ values ranging from 0.003 to 2.8 μM (Table 2).^61^ The mechanism of action has been convincingly characterized through resistance selection followed by validation using recombinant viruses. Collectively, these two series of 2C-compound 1 analogs demonstrated broad-spectrum antipicornaviral activity and represent promising starting points for further preclinical development against EV and RV infections.

### Virtual Screening-Derived 2C Inhibitors

The availability of crystal structures of the 2C proteins of EV-A71 and CV–B3, combined with reliable homology modeling tools, has enabled virtual screening campaigns targeting 2C. To elucidate key interactions between reported 2C inhibitors and enteroviral 2C, the Zhang group reconstructed 2C protein structures from 20 enteroviruses using the I-TASSER programs, followed by extensive docking studies with the 22 reported 2C inhibitors. However, due to uncertainties in computational predictions, additional pharmacological validation is required to confirm these findings.^159^

Using AtomNet@, a structure-based deep convolutional neural network virtual screening technology, White et al. screened millions of commercially available small molecules targeting the 2C-2C binding pocket originally occupied by the pocket binding domain at the C-terminal end of 2C monomer (Figure 2C).^33,160^ From the initial 77 compounds identified in silico, two hits, SJW-2C-14 and SJW-2C-69 (Table 2 and Figure 5A), showed activity in both an ATPase assay and cell-based CPE assays against EV-A71 in Vero cells. These compounds share a central bicyclic ring similar to ATP (highlighted in red, Figure 5A).^33^ To exclude ATP mimics, hit expansion was guided by a pharmacophore model derived from these VS hits, focusing on the 2-position of the benzimidazole ring and R1 and R2 substituents. Of 45 analogs, SJW-2C-184 (Table 2 and Figure 5A) exhibited the strongest antiviral activity in the CPE assay (EC_50_ = 5 μM). Further hit expansion yielded SJW-2C-227 (Table 2 and Figure 5A), the lead compound among 67 analogs, with an EC_50_ of 2.7 μM against EV-A7131. A mechanistic study using differential scanning fluorimetry (DSF) demonstrated direct binding between SJW-2C-227 and the EV-A71 2C protein. Interestingly, resistance to SJW-2C-69 mapped to a capsid protein mutation, VP1-T237N, rather than 2C. Serial passage of EV-A71 with SJW-2C-227 selected mutations VP4-K47E and 3A-R34W, neither of which was validated by reverse genetics.^33^ The interaction between 2C and capsid proteins during morphogenesis might account for this discrepancy.

**Figure 5:**
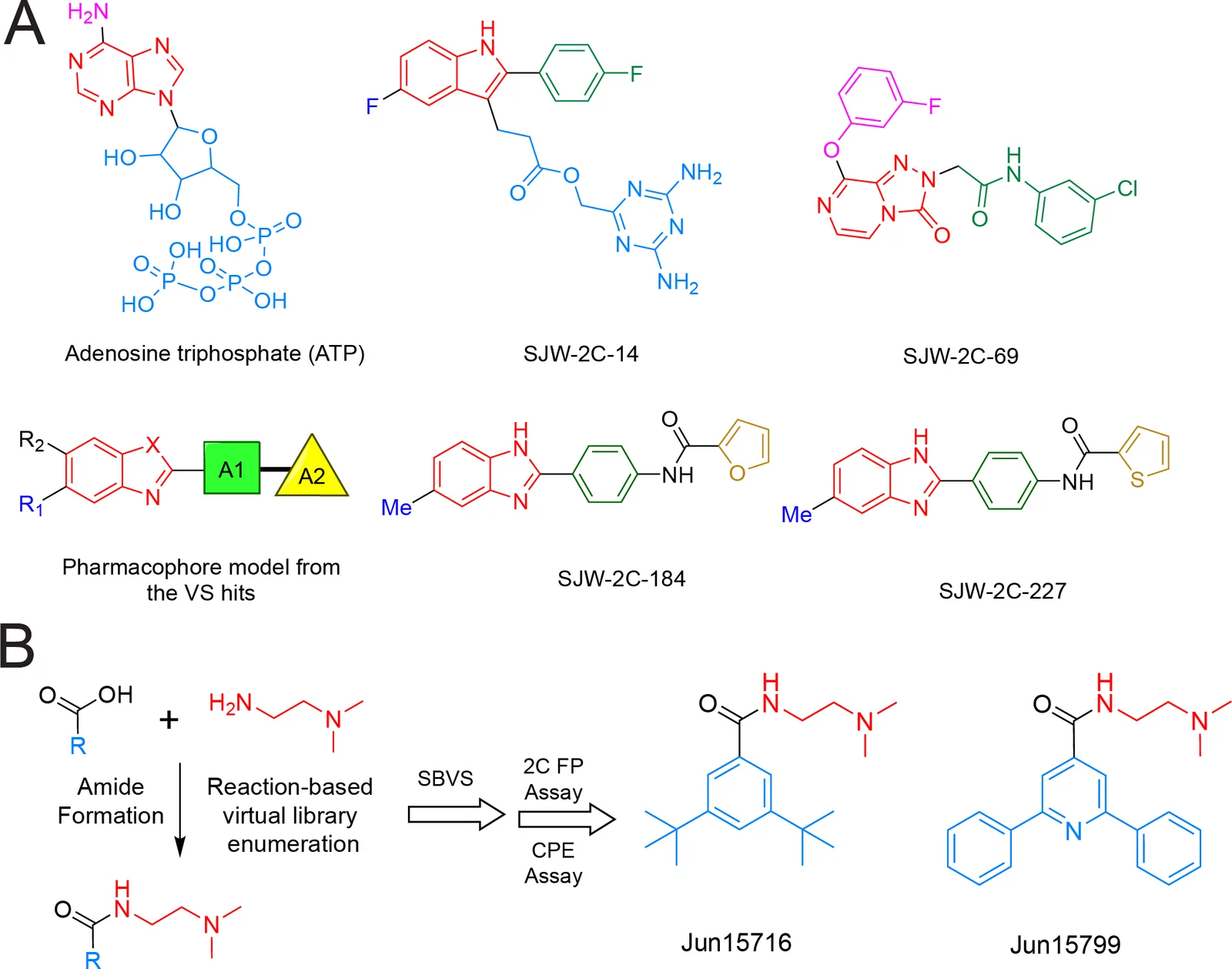
Identification of 2C inhibitors through virtual screening. (A) Comparison of the chemical structures of adenosine triphosphate (ATP) and screening hits, the pharmacophore model of the selected analogs of the hits, and the structures of the lead candidates as 2C inhibitors. (B) Discovery scheme of “Y”-shaped virtual screening hits as 2C inhibitors.

Our group also performed virtual screening targeting EV-D68 2C using a homology model generated by AlphaFold 3. We created a virtual library of 200,000 compounds using reaction-based combinatorial enumeration, synthesizing amide bonds between 2-dimethylaminoethylamine and diverse commercially available aromatic carboxylic acids.^40^ After filtering out compounds with unfavorable properties, docking-based virtual screening was conducted, followed by 2C fluorescence polarization (FP) assay and cell-based CPE assays. Two novel “Y”-shaped hits, Jun15716 and Jun15799 (Table 2 and Figure 5B), were validated as 2C inhibitors for further optimization and development.^40^

### Antiviral Peptide and Protein Targeting 2C

Beyond small-molecule inhibitors, peptides and protein-based agents that target 2C have demonstrated promising antiviral activity *in vitro* and, in some cases, *in vivo*. The C terminus of EV-A71 2C forms an amphipathic helix that inserts into a deep pocket of a neighboring 2C monomer and thereby mediates and stabilizes 2C homo-oligomerization (Figure 6).^35,161^ Exploiting this critical interaction site, a competitive peptide termed 2C-ligand (2CL, Table 2 and Figure 6) was designed on the basis of the EV-A71 2C C-terminal sequence.^161^ 2CL targets EV-A viruses, including EV-A71 and CV-A16, whereas B-2CL (Table 2 and Figure 6) targets EV-B viruses, including CV–B3 and echovirus 11.^161^ Both peptides incorporate the RNA-binding and pocket-binding segments of the C-terminal helix and are fused through a short linker to the TAT47–57 cell-penetrating peptide (CPP) (Figure 6).^161^ 2CL showed potent *in vitro* antiviral activity against EV-A71 and CV-A16 and demonstrated *in vivo* efficacy in protecting mice from lethal EV-A71 infection.^161^ Similarly, B-2CL inhibited replication of CV–B3 and echovirus 11 in cell culture.^161^ However, these antiviral peptides showed limited binding efficiency and suboptimal pharmacological properties, largely due to the appended CPP motif and the extended peptide sequence.^162^ Further optimization focused on shortening the peptide and removing the SGS–TAT segment.^162^ This effort produced three tetrapeptidomimetic inhibitors, 2CA-1 to 2CA-3, derived from the C-terminal Ala-Leu-Phe-Asn sequence of 2C and featuring diverse N-terminal capping groups with a free C-terminal amide (Figure 6).^162^ All three compounds showed single-digit micromolar antiviral activity against EV-A71.^162^ Among them, 2CA-1 displayed satisfactory oral bioavailability in mice, significantly reduced viral loads, and showed broad-spectrum antiviral activity against multiple enteroviruses, including CV-A6, CV-A16, CV–B3, echovirus 11, EV-D68, and rhinovirus (Table 2).^162^

**Figure 6:**
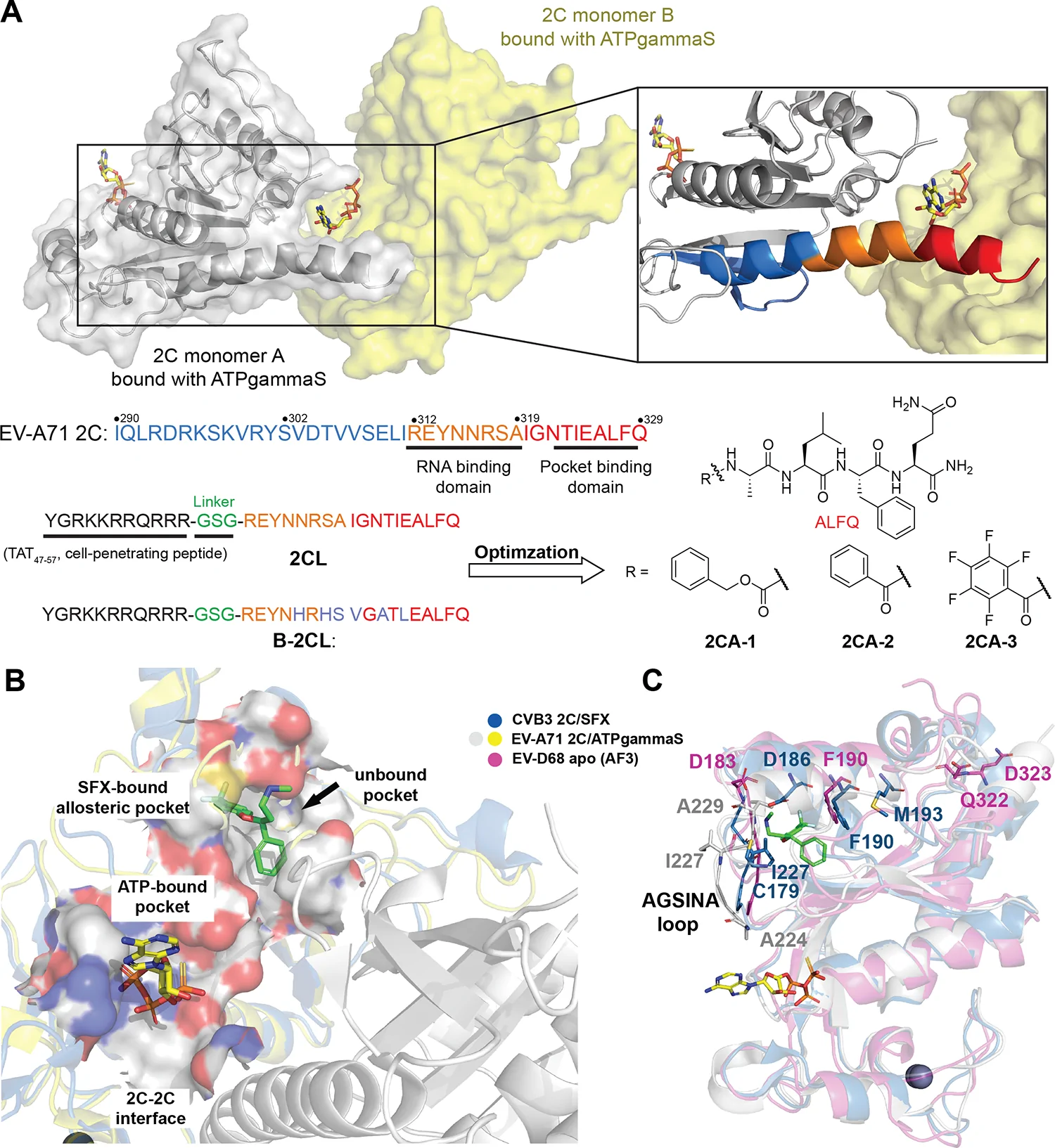
Identification of 2C inhibitors through rational peptide design and summary of reported binding pocket and resistant residues. (A) Design of an EV-A71 2C-targeting peptide (PDB ID: 5GRB). The amino acid sequence of the C-terminal domain, 2CL, and B-2CL. Chemical structural formulas of the three peptidomimetics, 2CA-1, 2CA-2, 2CA-3 obtained after further optimization. (B) The pockets of reported 2C inhibitors shown on EV-A71 2C (PDB ID: 5GRB) and CV-B3 2C (PDB ID: 6S3A). (C) Resistance mapping of the 2C proteins of CVB3, EV-A71, and EV-D68 with representative residues shown mutation.

These findings highlight that 2C-targeting peptides and peptidomimetics that disrupt 2C homo-oligomerization through the C-terminal helix-binding pocket represent a broad-spectrum antiviral strategy against picornaviruses and provide a strong foundation for further optimization. A related strategy has also been applied to FMDV 2C, for which peptides derived from the C-terminal pocket-binding loop rather than the helix were designed to disrupt 2C–2C interactions and prevent formation of functional hexameric assemblies.^38^

Radical S-adenosyl methionine domain-containing protein 2 (RSAD2), also known as viperin (Table 2), is an interferon-stimulated gene product with antiviral activity against multiple viruses.^163^ Structurally, viperin comprises an N-terminal domain, a C-terminal domain, and a highly conserved central radical SAM domain.^164^ Although viperin is a host antiviral factor involved in innate immune responses, it is discussed here because its antiviral activity against EV-A71 is mediated through direct interaction with the viral 2C protein.^165^ Wei et al. reported that viperin inhibits EV-A71 replication and is induced during infection both *in vitro* and *in vivo*.^164^ Its antiviral effect depends on interaction between EV-A71 2C and the N-terminal domain of viperin, which disrupts the function of the viral replication complexes.^164^

### Drug-Binding Pockets and Resistance Mapping of the 2C Protein

Although the precise binding sites of several screening hits, such as GuHCl, HBB, and loxapine succinate, remain unvalidated despite the emergence of resistance mutations in 2C, other inhibitors can be broadly classified into three categories: ATP-competitive inhibitors targeting the ATPase domain, allosteric inhibitors targeting the SFX-bound pocket, and protein–protein interaction inhibitors targeting pocket binding domain (Figure 6A,B).

The allosteric pocket of the 2C protein is characterized by rich aspartic acid residues near the pocket entrance (Figure 6B). Hydrogen-bond donors appear to be favorable binding motifs across the quinoline, pyrazolopyridine, Y-shaped symmetric, and SFX-based series of 2C allosteric inhibitors.^39,40,44,61,78^ In addition, the 2C allosteric pocket is branched, providing opportunities for further chemical elaboration. In the crystal structure, SFX occupied only part of the pocket. The introduction of additional aromatic substituents, which improved antiviral activity, was also observed during the optimization of the dibucaine series. These findings suggest that substantial unexplored chemical space remains available for targeting this allosteric pocket.

The catalytic ATP-binding pocket is reported to form only after a conformational rearrangement induced by the interaction between two neighboring 2C monomers, which may explain the impaired ATPase activity of truncated 2C monomers.^35–37^ The induced ATP-binding pocket is wide and shallow, located adjacent to the α6 helix and 2C-2C interface (Figure 6A,B). 2C inhibitors, such as metrifudil and N6-benzyladenosine, exert antiviral activity by competing for ATP binding within this pocket.

Studies of PPI inhibitors that disrupt 2C–2C interactions were mainly focused on the C-terminal protein binding domain on the α6 helix, although resistance mutation of loxapine succinate was reported at the N-terminal membrane-binding domain of HAV 2C. The 2CA peptidomimetic series has demonstrated moderate antiviral activity and a clear mechanism of action, whereas the precise targets of the SJW series identified by virtual screening require further clarification.^33,162^

Resistance mapping is one of the most important approaches for identifying potential 2C inhibitors and has revealed several residues that serve as hotspots for resistance mutations. Here, we provide a landscape of these residues across three of the most widely studied picornaviral 2C proteins, namely EV-D68, EV-A71, and CV-B3, offering insight into the interactions between these residues and reported 2C inhibitors (Figure 6C). It turns out that, except for the 224-AGSINA-229 loop (highlighted in ribbon representation), most resistance-associated residues are located on the α2 helix, such as D183, D186, F190, and M193, suggesting it is a critical structural component contributing to the formation of the allosteric pocket in 2C proteins (Figure 6C). Resistant mutations were also observed at residues on the C-terminal α6 helix of EV-D68 2C, such as Q322 and D323, which are involved in 2C oligomerization (Figure 6C).

### Assays for 2C Inhibitors Characterization

The identification and optimization of 2C inhibitors have been significantly hindered by the lack of robust and direct biochemical assays. As a member of the AAA+ ATPase family, 2C exhibits ATPase activity in its hexameric form.^36,37,51^ Consequently, biochemical characterization remains challenging due to difficulties in protein expression, purification, and assembly into functional oligomeric states.

Cell-based antiviral assays, such as CPE assays, combined with resistance-mapping studies, are widely used to evaluate the antiviral activity of 2C inhibitors and infer target engagement.^40,53,144–146^ These assays offer physiologically relevant readouts and can indirectly support target engagement. However, they do not provide direct evidence of binding to 2C. This limitation is highlighted by resistance studies, in which mutations associated with putative 2C inhibitors are sometimes found in viral proteins other than 2C, complicating target assignment.^146^ As a result, relying solely on phenotypic assays can lead to incorrect assignment of molecular targets. Moreover, these approaches are often time-consuming and labor-intensive, frequently requiring months to generate resistant viral strains and to validate drug sensitivity using recombinant systems.^166^

### Biophysical Binding Assays

Biophysical assays are widely used to directly quantify ligand–protein interactions in antiviral drug discovery. Techniques such as differential scanning fluorimetry (DSF) and isothermal titration calorimetry (ITC) have been applied to validate binding between small molecules and the 2C protein (Figure 7A).^39,40,53^ Despite their utility, these methods are generally low-throughput and resource-intensive, limiting their application in large-scale screening. Furthermore, they require high-quality, stable protein preparations, which are not always readily available for 2C.^51^

**Figure 7:**
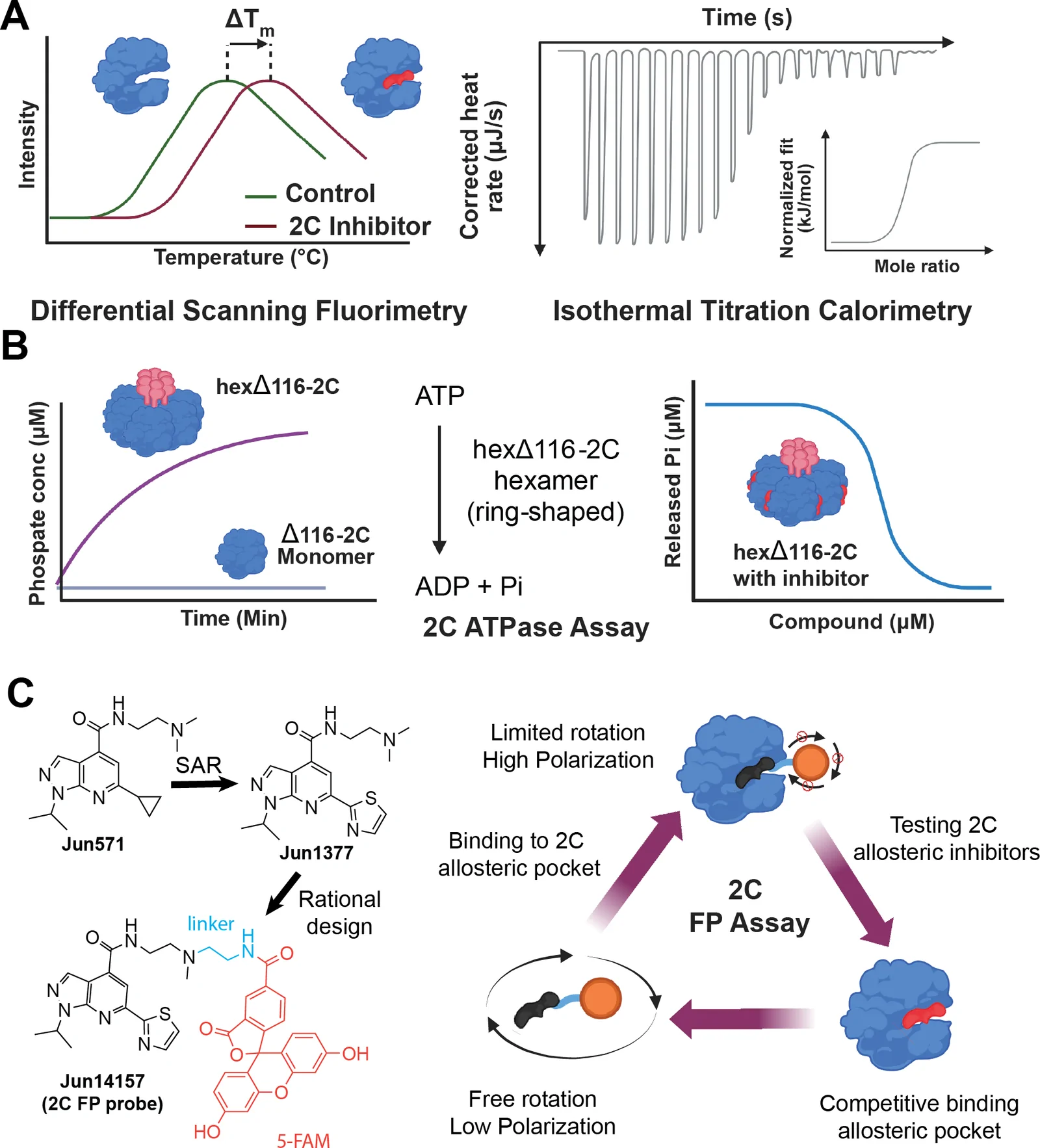
Assays for evaluating inhibitors targeting the Picornavirus 2C protein. (A) Biophysical assays (DSF, ITC) provide direct evidence of ligand–2C binding. (B) 2C ATPase assays measure phosphate release using engineered hexameric 2C constructs. (C) 2C FP assays enable direct, quantitative measurement of binding. Figure created with BioRender.

### ATPase-Based Biochemical Assay

Efforts to evaluate 2C inhibitors initially relied on ATP hydrolysis assays that measure inorganic phosphate production as a readout for ATPase activity, often using an engineered hexameric construct (hexΔ116–2C).^37,44^ These assays provide a direct functional readout of enzymatic activity and have been used to characterize classical inhibitors such as guanidine hydrochloride and fluoxetine, as well as their derivatives (Figure 7B).^37,44^

However, several limitations restrict their utility. First, ATPase activity of monomeric 2C is extremely low and highly dependent on oligomerization, resulting in poor signal-to-noise ratios.^37,40^ Second, full-length 2C tends to aggregate during expression due to its amphipathic N-terminus, whereas truncated constructs are typically soluble but predominantly monomeric.^51^ Third, ATP-competitive or ATP-mimicking compounds may generate false-positive results, complicating hit validation.^33^ Finally, these assays do not directly confirm binding at specific allosteric sites, which are increasingly recognized as key druggable regions for many broad-spectrum 2C inhibitors.^37^

### Fluorescence Polarization (FP)-Based Binding Assay

To meet the need for direct, high-throughput binding assays, fluorescence polarization (FP)–based platforms have recently emerged as a powerful solution. In this approach, a free (unbound) FP probe exhibits low polarization due to rapid rotational diffusion, whereas binding to 2C slows its motion, increasing the polarization signal. Competitive displacement of the bound probe by potent inhibitors correspondingly reduces the FP signal (Figure 7C). Leveraging this principle, a fluorescently labeled probe, Jun14157—derived from a pyrazolopyridine-based 2C inhibitor scaffold—was developed and applied to quantitatively measure the binding affinity of EV-D68 2C inhibitors (Figure 7C).^39,40^ FP assays offer several advantages, including high sensitivity and compatibility with high-throughput screening. The robustness of HTS with FP assay is typically evaluated using the *Z*′ factor, a statistical parameter that reflects assay quality and reproducibility. Importantly, FP-based assays enable quantitative determination of binding affinity (*K_i_* values) and provide a direct measure of target engagement.^40^ One limitation of the FP assay is that, due to the binding mode of the pyrazolopyridine probe, the assay is primarily applicable to the evaluation of 2C allosteric inhibitors. In addition, the FP assay serves as a binding assay and therefore cannot directly assess inhibition of 2C catalytic activity, despite the strong correlation observed between FP binding affinity and antiviral activity. Consequently, complementary biochemical assays, such as ATPase assays with hexameric 2C proteins, should be incorporated to cross-validate putative 2C allosteric inhibitors.

## Conclusion and Future Perspectives

Picornaviruses continue to pose a substantial public health burden, with large numbers of infections reported each year. An unmet need, therefore, remains for effective antiviral therapeutics. However, no FDA-approved drugs are currently available. Considerable progress has nevertheless been made in the discovery of antipicornaviral agents that target both structural and nonstructural proteins required for viral replication. Among the nonstructural proteins, 2C is highly conserved and performs multiple essential functions during viral replication and virus–host interactions. These features make 2C an attractive target for broad-spectrum antiviral development. In this review, we highlight 2C as a promising antiviral target by summarizing its structural biology, biological functions, and currently reported inhibitors.

Structurally diverse 2C inhibitors have been reported, spanning a wide range of potencies and antiviral spectra. Together, these chemotypes establish a solid foundation for further lead optimization and translational development.^67,68^ The availability of the X-ray crystal structure of CV-B3 2C in complex with fluoxetine provides a critical structural framework for rational, structure-based design, enabling detailed interrogation of ligand binding modes, allosteric pockets, and resistance hotspots.^37^ Encouragingly, a recently reported 2C inhibitor, Jun6504, demonstrated preclinical *in vivo* antiviral efficacy in an EV-D68 neurotropic mouse model.^40^ This result not only highlights the tractability of 2C for small-molecule intervention but also provides strong pharmacological validation of 2C as a viable and promising antiviral drug target.

In addition to advances in inhibitor discovery, the development of robust, mechanism-informed assays is critical to accelerating 2C-targeted antiviral research. Current validation strategies rely heavily on CPE assays and follow-up resistant-mapping studies; however, this approach is time-consuming and not 2C-target-specific. Biophysical techniques, such as DSF and ITC, provide direct evidence of ligand binding but remain low-throughput and resource-intensive.^39,40,53^ ATPase assays often suffer from a low signal-to-background ratio and dependence on oligomerization state.^37,44^ Recent developments in FP-based binding assays represent an important advance, enabling direct, quantitative, and high-throughput measurement of ligand–2C interactions with robust assay performance.^40^ The continued evolution of assay technologies will play a central role in bridging the gap between *in vitro* biochemical activity or binding affinity and antiviral activity, ultimately facilitating the discovery of mechanistically validated 2C inhibitors.

Several future directions may accelerate the development of 2C-targeted antivirals. First, although 2C is highly conserved among picornaviruses, not all reported 2C inhibitors display broad-spectrum antiviral activity. For example, metrifudil and N6-benzyladenosine were active against EV-A71 but inactive against PV,^141^ fluoxetine showed preferential activity against EV-B and EV-D species,^120,152^ and JX040 inhibited EV-A71 and CV–B3 but not PV-1.^157^ The lack of broad-spectrum antiviral activity is further supported by the distinct profiles of resistance and cross-resistance reported across different chemotypes. For example, MRL-1237 showed cross-resistance with GuHCl but not with enviroxime,^135^ and different inhibitor classes selected distinct resistance substitutions in 2C.^120,135,141,151^ These observations strongly suggest that individual 2C inhibitors engage different binding sites or binding modes. Future medicinal chemistry efforts should therefore prioritize inhibitors that engage the most conserved and functionally constrained regions of 2C to maximize the likelihood of broad-spectrum antiviral activity. Alternatively, 2C inhibitors with nonoverlapping resistance profiles can be developed as complementary agents for use in combination therapy.

Second, the structural biology of inhibitor-bound 2C needs substantial expansion. Although several crystal structures of 2C have now been reported, cocomplex structures with bona fide inhibitors remain limited, with S-fluoxetine and compound 53 in CV–B3 2C representing key examples.^37,44^ For many additional chemotypes, including compounds active against EV-D68 and other enteroviruses, binding models still rely heavily on homology modeling, docking, and molecular dynamics simulations.^40^ Given the remarkable structural diversity of reported 2C inhibitors, additional cocrystal or cryo-EM structures will be essential for defining binding modes at high resolution and guiding future structure-based design.

Third, the 2C–2C dimer or oligomerization interface represents an emerging and underexploited drug target. Structural studies have shown that the C-terminal helix or pocket-binding loop mediates critical intersubunit interactions required for functional 2C assembly.^35,36,38^ Peptides, peptidomimetics, and related inhibitors that disrupt this interface have already demonstrated proof-of-concept antiviral activity, but their potency, binding efficiency, and pharmacokinetic properties still require further optimization.^161,162^

Fourth, because 2C engages multiple host factors involved in membrane remodeling, replication-organelle biogenesis, and nucleotide metabolism, targeting selected 2C–host protein interactions may offer an additional antiviral strategy. Representative examples discussed above include interactions with reticulon 3, p97/VCP, HSP70 family proteins, CAD, Beclin1, and vimentin.^25,34,71–85^

Beyond these directions, additional opportunities include developing full-length, ligand-bound hexameric 2C structural models, incorporating pan-enterovirus resistance profiling early in lead optimization, and exploring targeted degradation strategies such as PROTACs or molecular glues. The latter approach is supported by the availability of 2C inhibitors and by the successful development of the fluorescent polarization probe Jun14157, which suggests that linker installation can preserve 2C binding (Figure 7C).^40,167^ At the same time, because 2C participates in complex virus–host interactions, degrader-based strategies will require careful evaluation of selectivity and the risk of perturbing host homeostasis. Besides, there will also be a significant technical barrier for the evaluation of 2C PROTAC, due to its properties of transient expression, membrane-association, and oligomerization.

Collectively, 2C remains one of the most promising antiviral targets in the picornavirus field. This review summarizes current understanding of 2C structure in monomeric and hexameric forms, the relationships between its domains and biological functions, and the diverse classes of reported 2C-targeting inhibitors. Although substantial progress has been made, translation of these advances into clinically useful antivirals will require continued integration of structural biology, medicinal chemistry, virology, and pharmacology. We anticipate that the knowledge compiled here will help guide the next generation of 2C-directed antiviral discovery efforts.

## List of Abbreviations

2C-Δ116 (hexΔ116–2C): engineered hexameric 2C construct
3B (VPg): picornavirus 3B viral protein genome-linked
3Cpro: picornavirus 3C protease
3Dpol: picornavirus 3D RNA-dependent RNA polymerase
AFM: acute flaccid myelitis
AH: amphipathic helix
ATP: adenosine triphosphate
ATP-γ-S: adenosine 5′-*O*-(3-thiotriphosphate)
AUC: area under the curve
CAD: carbamoyl phosphate synthetase/aspartate transcarbamylase/dihydroorotase
CPP: cell-penetrating peptide
CPE: cytopathic effect
CV: coxsackievirus
CV-A: coxsackievirus A
CV–B: coxsackievirus B
DMTA: design–make–test–analyze
DSF: differential scanning fluorimetry
EC_50_: half-maximal effective concentration
EMCV: encephalomyocarditis virus
ER: endoplasmic reticulum
FMDV: foot-and-mouth disease virus
FP: fluorescence polarization
FRET: fluorescence resonance energy transfer
GuHCl: guanidine hydrochloride
HAV: hepatitis A virus
HBB: 2-(α-hydroxybenzyl)-benzimidazole
HTS: high-throughput screening
IFN-β: interferon β
IKK: IκB kinase
ITC: isothermal titration calorimetry
*K_i_*: inhibition constant
MDA5: melanoma differentiation-associated protein 5
MHC I: major histocompatibility complex class I
NF-κB: nuclear factor kappa B
NMRI: Naval Medical Research Institute
ORF: open reading frame
PBD: pocket-binding domain
PBL: protein-binding loop
PK: pharmacokinetics
PV: poliovirus
RIG-I: retinoic acid-inducible gene I
RNA: ribonucleic acid
RdRp: RNA-dependent RNA polymerase
RV: rhinovirus
RT-qPCR: reverse transcription quantitative polymerase chain reaction
SAR: structure–activity relationship
SF3: superfamily 3 (helicase-like proteins)
SFX: (S)-fluoxetine
UTR: untranslated region
VS: virtual screening
ZFER: zinc-finger equivalent region
